# Meningeal Lymphatic LTBR Deficiency Links Spinal Cord Injury to Secondary Brain Pathology

**DOI:** 10.1002/advs.78206

**Published:** 2026-10-11

**Authors:** Yi Chen, Yu Tang, Chengpeng Hu, Xiaoyan Duan, LingXia Min, Boya Cong, Chao Ma, Mingliang Tan, Ya Luo, Pengfei Wei, Minpeng Xu, Zhou Feng, Jingming Hou

**Affiliations:** ^1^ Department of Rehabilitation Southwest Hospital Third Military Medical University (Army Medical University) Chongqing China; ^2^ School of Biological Science and Medical Engineering State Key Laboratory of Digital Medicine Southeast University Nanjing Jiangsu China; ^3^ Academy of Medical Engineering and Translational Medicine Tianjin University Tianjin China

**Keywords:** DTI‐ALPS index, lymphatic system, lymphotoxin beta receptor, neuroinflammation, secondary neural injury

## Abstract

Spinal cord injury (SCI) induces secondary brain pathology, but the mechanisms linking spinal and brain injury remain unclear. Here, we identify meningeal lymphatic dysfunction as a contributor to secondary brain pathology after SCI. In mice, meningeal lymphatic vessels (mLVs) progressively deteriorated from 14 to 56 days post‐injury, accompanied by glymphatic dysfunction. In patients with SCI, diffusion tensor imaging along the perivascular space (DTI‐ALPS) revealed impaired glymphatic function, which correlated with motor cortical and hippocampal atrophy. mLVs ablation exacerbated neuroinflammation, neuronal loss, impaired hippocampal neurogenesis, and motor and cognitive deficits, whereas VEGF‐C‐mediated lymphatic restoration ameliorated these abnormalities. Integrated single‐cell and bulk transcriptomics identified reduced LTBR expression in meningeal lymphatic endothelial cells (LECs) after SCI. *Ltbr* knockdown in LECs further exacerbated SCI‐induced lymphatic dysfunction and secondary brain pathology, whereas LEC‐specific *Ltbr* overexpression promoted lymphatic repair and improved neurological outcomes. In human LECs, LTBR enhanced proliferation, migration, and tube formation, with PI3K‐AKT signaling potentially contributing to these effects. These findings establish meningeal lymphatic dysfunction as an important component of secondary brain pathology after SCI and identify LTBR as a potential regulator of lymphatic repair.

## Graphical abstract

Spinal cord injury (SCI) induces remote brain alterations beyond the injured spinal cord. This study links dysfunction of meningeal lymphatic vessels (mLVs) to neuroinflammation and neuronal injury after SCI. LTBR‐mediated lymphatic restoration improves meningeal lymphatic integrity and neurological outcomes, highlighting LTBR as a potential regulator of lymphatic remodeling after SCI. Created with BioRender.com under a license.

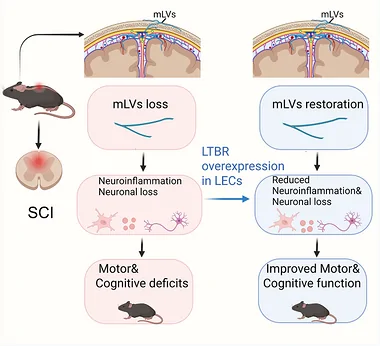

## 1 Introduction

Brain structural and functional impairments secondary to spinal cord injury (SCI) represent a significant yet frequently overlooked complication. These remote brain pathologies not only hinder the recovery of motor and sensory functions [1], but also contribute to cognitive and affective deficits [2, 3]. Evidence indicates that over 60% of SCI patients develop cognitive impairment, including deficits in attention, memory, and executive function [4]. Although advances in tissue engineering and robotic exoskeletons show considerable promise for motor rehabilitation, such interventions do not address SCI‐related brain disorders [5]. Thus, elucidating the mechanisms underlying secondary brain injury post‐SCI is essential for developing comprehensive therapeutic strategies.

Our previous work identified pronounced brain atrophy in patients with SCI [6], an observation that has been corroborated by independent studies [7, 8]. This structural degeneration correlates with neurological dysfunction: the extent of primary motor cortex (M1) atrophy strongly predicts motor recovery [1]. Further mechanistic investigation revealed that SCI triggers robust neuroinflammation within the cerebral cortex, culminating in neuronal death [9]. Similar neuroinflammatory changes occur in the hippocampus, contributing to neuronal injury and functional impairment [10, 11, 12]. Thus, widespread neuroinflammation emerges as a pivotal mechanism mediating post‐SCI brain damage, yet the mechanisms initiating and sustaining this response remain unknown.

The recent discovery of meningeal lymphatic vessels (mLVs) has revolutionized our understanding of brain homeostasis and disease pathogenesis [13]. These structures, integral to the glymphatic‐lymphatic system, facilitate cerebrospinal fluid (CSF) drainage and immune cell trafficking between the central nervous system (CNS) and peripheral immunity [14, 15]. Impaired meningeal lymphatic function contributes to neuroinflammation and neurological dysfunction in various CNS disorders, including neurodegenerative diseases, neuroinflammatory conditions, and traumatic brain injury [16, 17, 18]. Dysfunctional mLVs compromise CSF clearance, leading to neurotoxic waste accumulation and sustained inflammation within the brain parenchyma [15, 19]. However, whether SCI affects mLVs integrity and function, and whether such changes trigger secondary brain injury, remains unexplored.

In the present study, we demonstrate that SCI induces structural and functional damage to mLVs, impairing CSF drainage, promoting neuroinflammation in the remote cortex and hippocampus, and ultimately contributing to motor and cognitive deficits. Using human imaging, mouse models, single‐cell and transcriptomic sequencing analyses, and gain‐ and loss‐of‐function approaches, we further identify lymphotoxin beta receptor (LTBR) in meningeal lymphatic endothelial cells (LECs) as a key regulator of meningeal lymphatic repair after SCI. Our findings establish mLVs dysfunction as a critical pathological mechanism linking SCI to remote brain injury and identify LTBR as a potential regulator of meningeal lymphatic repair and a candidate target for future intervention strategies.

## 2 Results

### 2.1 SCI Impairs Meningeal Lymphatic and Brain Glymphatic Function

To investigate the brain lymphatic dynamics after SCI, we established a T9 spinal cord contusion model in mice. At 1, 7, 14, 28, and 56 days post‐injury (dpi), Alexa Fluor 647‐conjugated ovalbumin (OVA‐647) was injected into the cisterna magna, and tracer distribution was assessed in the brain and deep cervical lymph nodes (dCLNs) 2 h later. Tracer drainage to the dCLNs measured using in vivo imaging (Figure 1A,B) and histological quantification (Figure 1C,D) showed a significant reduction in SCI group starting at 14 dpi and persisting through 56 dpi, revealing sustained meningeal lymphatic dysfunction throughout the 14–56 dpi observation period. Consistent with these functional changes, lymphatic vessel endothelial hyaluronan receptor 1 (LYVE‐1) immunostaining revealed a progressive reduction in dural lymphatic vessel coverage beginning at 14 dpi and persisting to 56 dpi (Figure 1E,F). Similarly, compared with the sham group, tracer influx into brain parenchyma was significantly reduced beginning at 28 dpi and remained decreased through 56 dpi (Figure 1G,H), indicating impaired glymphatic function. To assess glymphatic‐related function in patients with SCI, we performed diffusion tensor imaging along the perivascular space (DTI‐ALPS) in SCI patients and healthy controls (Figure 1I). The ALPS index was significantly lower in the SCI group than in controls (Figure 1J), suggesting impaired glymphatic‐related function following SCI. We further quantified regional brain volumes and found significant atrophy of the precentral gyrus and hippocampus in SCI patients (Figure 1K,L). Correlation analysis revealed a significant positive correlation between the ALPS index and the volumes of both brain regions in SCI patients (Figure 1M,N), indicating an association between impaired glymphatic‐related function and structural brain alterations after SCI. Together, these demonstrate impairment of meningeal lymphatic drainage and glymphatic‐related tracer transport after SCI.

**FIGURE 1:**
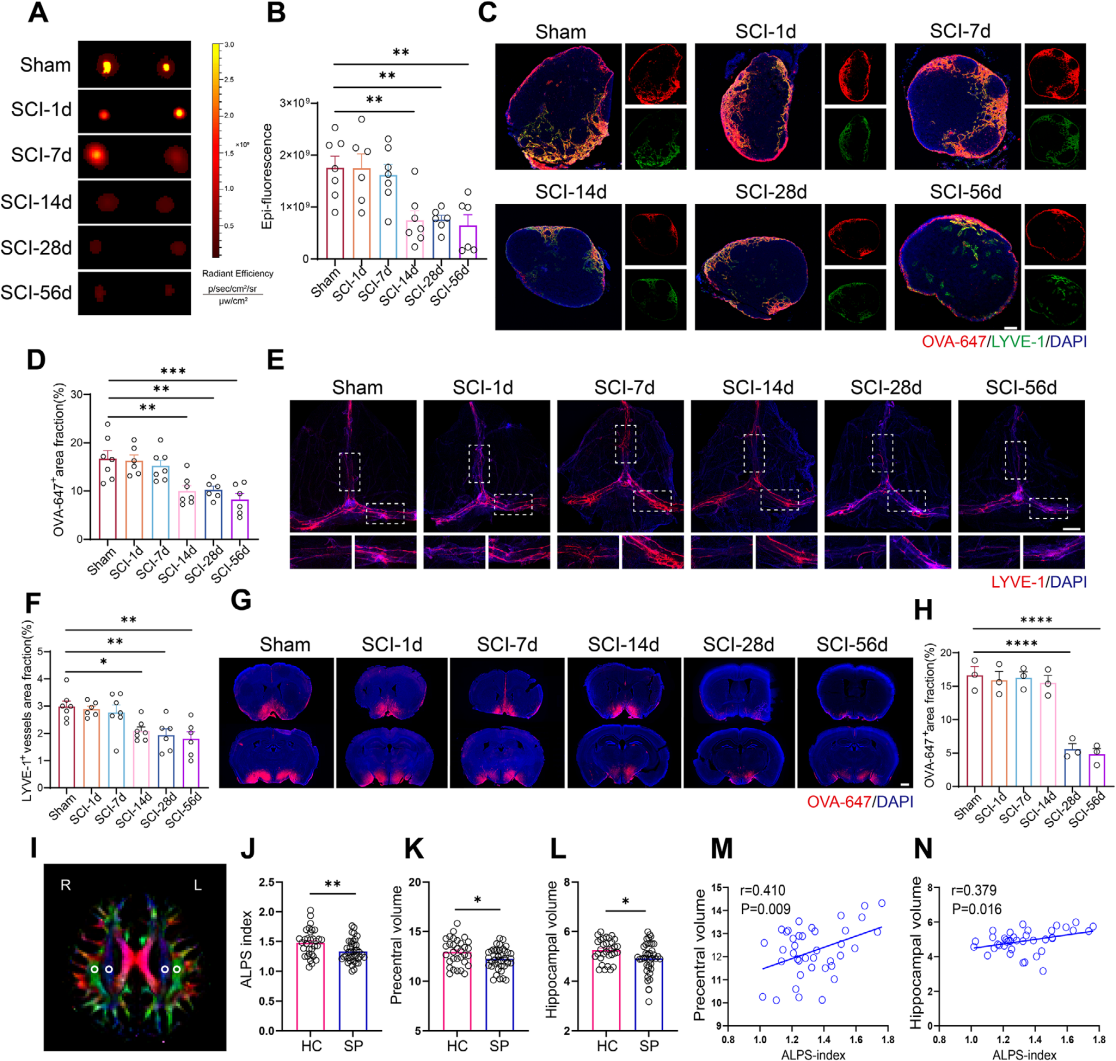
The effects of SCI on meningeal lymphatic and brain glymphatic function. (A, B) Representative in vivo bioluminescence images and quantification of fluorescence intensity of dCLNs at the indicated time points after SCI (n = 6‐7 mice per group). (C) Representative immunofluorescence images of dCLNs sections stained for OVA‐647 (red), LYVE‐1 (green), and 4′,6‐diamidino‐2‐phenylindole (DAPI, blue) at the indicated time points after SCI (n = 6‐7 mice per group). Scale bar, 200 µm. (D) Quantification of OVA‐647^+^ area fraction in dCLNs sections at the indicated time points after SCI (n = 6‐7 mice per group). (E, F) Representative immunofluorescence images and quantification of LYVE‐1^+^ vessel area fraction of the dura mater stained for LYVE‐1 (red) and DAPI (blue) at the indicated time points after SCI (n = 6‐7 mice per group). (G, H) Representative coronal brain sections and quantification of OVA‐647^+^ area fraction at 2 h after intracisternal injection of OVA‐647 in sham‐operated mice and mice at 1, 7, 14, 28 and 56 day post‐SCI (n = 3 mice per group). Scale bar, 1000 mm. Statistical analysis was performed by one‐way ANOVA followed by Dunnett's multiple comparisons test. (I) Representative diffusion tensor imaging along the perivascular space (DTI‐ALPS) map, with ROI (white circles) placed in the perivascular space of the bilateral hemispheres (R: right hemisphere; L: left hemisphere). (J) Statistical analysis for ALPS index between SCI patients and healthy controls (HC, n = 32 participants; HP, n = 40 participants). (K, L) Volumetric analysis of the precentral gyrus and hippocampus in SCI patients and healthy controls. Statistical analysis was performed by unpaired two‐tailed Student's t‐test. (M, N) Pearson correlation analysis of DTI‐ALPS index with precentral gyrus and hippocampus in SCI patients. Scale bar,1000 µm. Data are presented as mean ± SEM. ^*^ *p* < 0.05, ^**^ *p* < 0.01, ^***^ *p* < 0.001, ^****^ *p* < 0.0001.

### 2.2 SCI Leads to Neurological Deficits and Remote Secondary Brain Pathology

To determine whether SCI‐induced lymphatic dysfunction is accompanied by sustained brain pathology, we evaluated neurological function, neuroinflammation, neuronal integrity, and transcriptomic profiles in mice at 56 dpi. Motor function, assessed longitudinally with the Basso Mouse Scale (BMS), remained markedly impaired in SCI mice throughout the observation period compared with sham controls (Figure S1A). Similarly, motor evoked potential (MEP) recordings at 56 dpi revealed prolonged latency and reduced amplitude in the SCI group (Figure S1B–D), indicating impaired corticospinal conduction. Cognitive function was evaluated using the novel object recognition (NOR) test and the Y‐maze test. Compared with the sham group, SCI mice exhibited a significant reduction in the object recognition index (Figure S1E) and fewer entries into and less time spent in the novel arm of the Y‐maze (Figure S1F–H), reflecting hippocampus‐dependent memory deficits. Neuroinflammation in the cortex and hippocampus was subsequently assessed via examining microglial activation and pro‐inflammatory cytokine levels. Enzyme‐linked immunosorbent assay (ELISA) demonstrated elevated levels of tumor necrosis factor‐alpha (TNF‐α) and interleukin‐1 beta (IL‐1β) in the same brain regions of SCI mice (Figure S1I,J), confirming an ongoing neuroinflammatory state. Consistently, immunofluorescence staining for ionized calcium‐binding adapter protein 1 (Iba1, a microglial marker) revealed a marked increase in microglial density in both regions of SCI mice (Figure S1K,L). Neuronal pathology was evaluated by Nissl staining and doublecortin (DCX) staining. Nissl staining further revealed decreased neuronal density in both the cortex and hippocampus of SCI mice (Figure S1M,N), suggesting that chronic neuroinflammation coincides with neuronal loss. A significant reduction in DCX‐positive cells was observed in the hippocampal dentate gyrus of SCI mice (Figure S1O,P), indicating impaired neurogenesis.

To further investigate the molecular mechanisms underlying these pathological changes, we performed RNA sequencing (RNA‐seq) analysis on the motor cortex and hippocampus at 56 dpi. Differential expression analysis revealed distinct transcriptional profiles between groups (Figure S2A,B), with the motor cortex and hippocampus exhibiting similar transcriptional alterations. Kyoto Encyclopedia of Genes and Genomes (KEGG) pathway enrichment analysis highlighted significant upregulation of gene sets associated with neuroinflammation and tissue remodeling after SCI, including NF‐kappa B signaling pathway, TGF‐beta signaling pathway, ECM‐receptor interaction, and Neuroactive ligand‐receptor interaction (Figure S1Q–T). Gene Ontology (GO) functional enrichment analysis also revealed enrichment in biological processes related to neuropeptide signaling, cell adhesion, neuronal differentiation and apoptosis, and calcium ion transport (Figure S2C,D). Additionally, Gene Set Enrichment Analysis (GSEA) confirmed significant enrichment of gene sets involved in inflammatory responses, DNA repair, synaptic plasticity, and impaired neuronal differentiation (Figure S2E,F). Collectively, these results demonstrate that SCI induces sustained neuroinflammation, neuronal loss, and impaired neurogenesis in the remote brain regions, accompanied by molecular programs consistent with progressive secondary brain injury.

### 2.3 Meningeal Lymphatic Ablation Exacerbates Neurological Deficits and Secondary Brain Pathology After SCI

To test whether meningeal lymphatic dysfunction contributes causally to secondary brain injury after SCI, we performed targeted mLVs ablation before SCI by intracisternal injection of Visudyne followed by photoactivation (Figure S3A). Compared to non‐ablated SCI controls, mLVs‐ablated mice exhibited significantly reduced tracer drainage to the dCLNs at 56 dpi, as measured by in vivo fluorescence imaging and histological analysis (Figure S3B–E). Consistently, immunofluorescence staining confirmed a marked reduction in mLVs coverage area in ablated mice (Figure S3F,G). mLVs ablation also reduced tracer influx into the brain parenchyma (Figure S3H,I), indicating that disruption of meningeal lymphatic outflow further compromises glymphatic exchange after SCI.

Behavioral tests were conducted at various time points post‐SCI to evaluate motor and cognitive abilities across groups (Figure S3A). BMS scores were significantly lower in the ablation group from 14 to 56 dpi compared to non‐ablated SCI controls (Figure S4A). MEP recordings at 56 dpi showed prolonged latency and reduced amplitude in the ablation group (Figure S4B–D). In behavioral tests, mLVs ablation worsened performance in both the NOR and Y‐maze tasks (Figure S4E–H), indicating exacerbated cognitive impairment.

Neuropathological examination at 56 dpi revealed that mLVs ablation amplified SCI‐induced brain pathology. TNF‐α and IL‐1β concentrations were further increased in the cortex and hippocampus (Figure S4I,J), and the density of Iba1‐positive microglia was markedly higher (Figure S4K,L). mLVs ablation also increased neuronal loss in the cortex and hippocampus and further reduced the number of DCX‐positive cells in the dentate gyrus (Figure S4M–P). These results support a causal role for meningeal lymphatic dysfunction in promoting glymphatic failure, neuroinflammation, neuronal injury, and neurological deficits after SCI.

### 2.4 VEGF‐C‐Mediated Lymphatic Restoration Mitigates Secondary Brain Pathology After SCI

Given the established role of vascular endothelial growth factor C (VEGF‐C) in promoting lymphatic growth and regeneration [20], we investigated whether enhancing meningeal lymphatic repair could reverse SCI‐induced secondary brain injury. Twenty‐eight days prior to SCI modeling, AAV1‐VEGF‐C or a control AAV1‐GFP virus was administered via intracisternal magna injection (Figure 2A). *Vegf‐c* overexpression in the dura mater was confirmed by real‐time quantitative PCR (qPCR) (Figure 3B). At 56 dpi, VEGF‐C treatment increased OVA‐647 drainage to the dCLNs, as shown by both in vivo imaging and histological quantification (Figure 2C–F). LYVE‐1 staining also revealed greater mLVs coverage in AAV1‐VEGF‐C treated mice than in controls (Figure 2G,H). Consistent with enhanced lymphatic drainage, tracer influx into the brain parenchyma was also increased (Figure 2I,J), reflecting improved glymphatic function.

**FIGURE 2:**
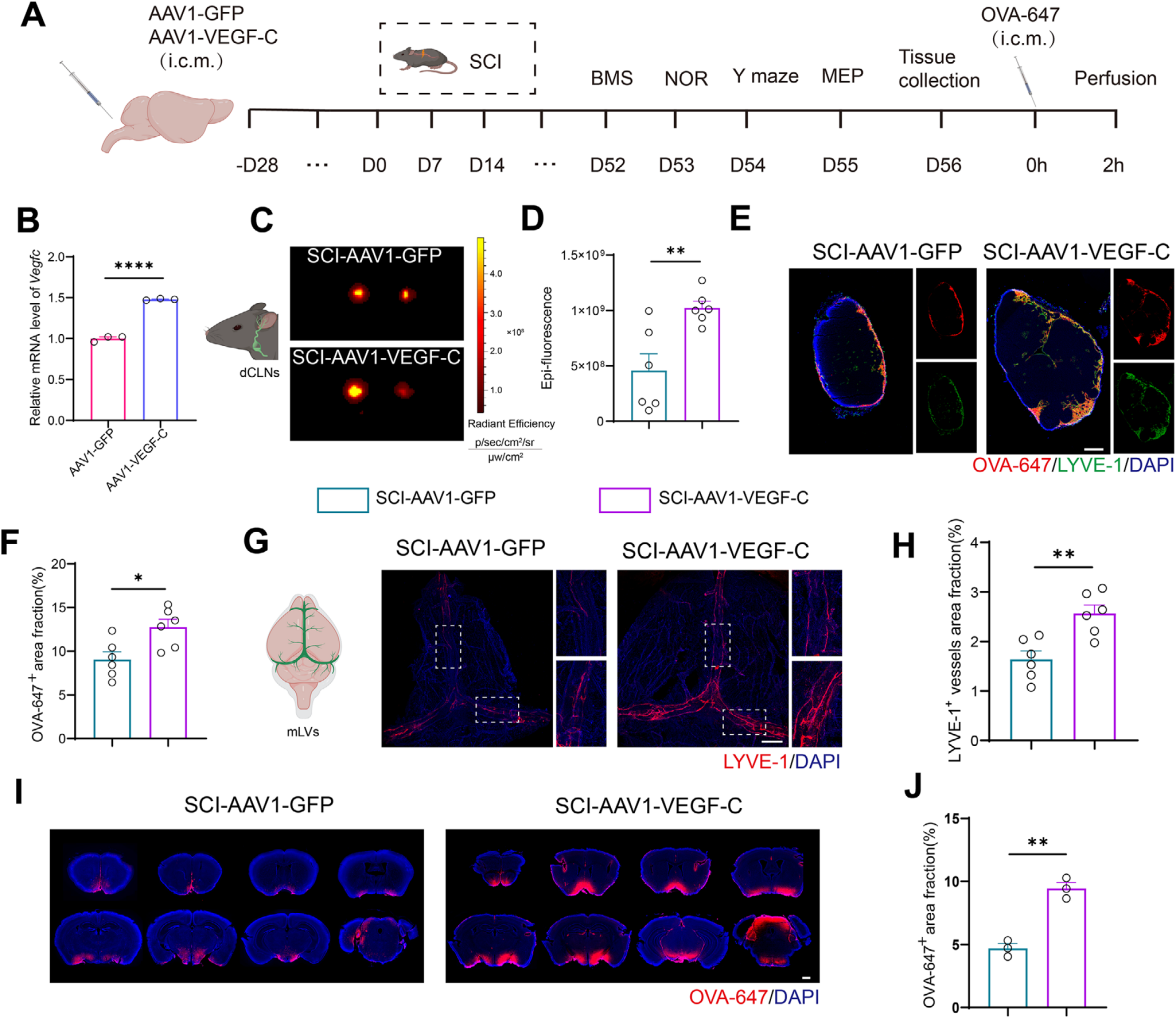
The effects of VEGF‐C treatment on mLVs function and CSF influx after SCI. (A) Schedule of treatments and behavior tests of SCI mice. (B) Relative mRNA expression of *Vegf‐c* in the dura mater of mice injected (i.c.m) with AAV1‐GFP or AAV1‐VEGF‐C (n = 3 mice per group). (C, D) Representative in vivo fluorescence images and quantification of fluorescence intensity of dCLNs in SCI mice injected with AAV1‐GFP or AAV1‐VEGF‐C at 56 dpi (n = 6 mice per group). (E) Representative immunofluorescence images of dCLNs sections stained for OVA‐647 (red), LYVE‐1 (green), and DAPI (blue) in SCI mice injected with AAV1‐GFP or AAV1‐VEGF‐C at 56 dpi. Scale bar, 200 µm. (F) Quantification of OVA‐647^+^ area fraction in dCLNs sections from SCI mice injected with AAV1‐GFP or AAV1‐VEGF‐C at 56 dpi (n = 6 mice per group). (G, H) Representative immunofluorescence images and quantification of LYVE‐1^+^ vessel area fraction in the dura mater of SCI mice injected with AAV1‐GFP or AAV1‐VEGF‐C at 56 dpi (n = 6 mice per group). Scale bar,1000 µm. (I, J) Representative coronal brain sections and quantification of OVA‐647^+^ area fraction showing CSF influx into the brain parenchyma in SCI mice injected with AAV1‐GFP or AAV1‐VEGF‐C at 56 dpi (n = 3 mice per group). Scale bar, 1000 mm. Statistical significance was determined by unpaired two‐tailed Student's t‐test. Data are presented as mean ± SEM. ^*^ *p* <0.05, ^**^ *p* < 0.01, ^****^ *p* < 0.0001.

**FIGURE 3:**
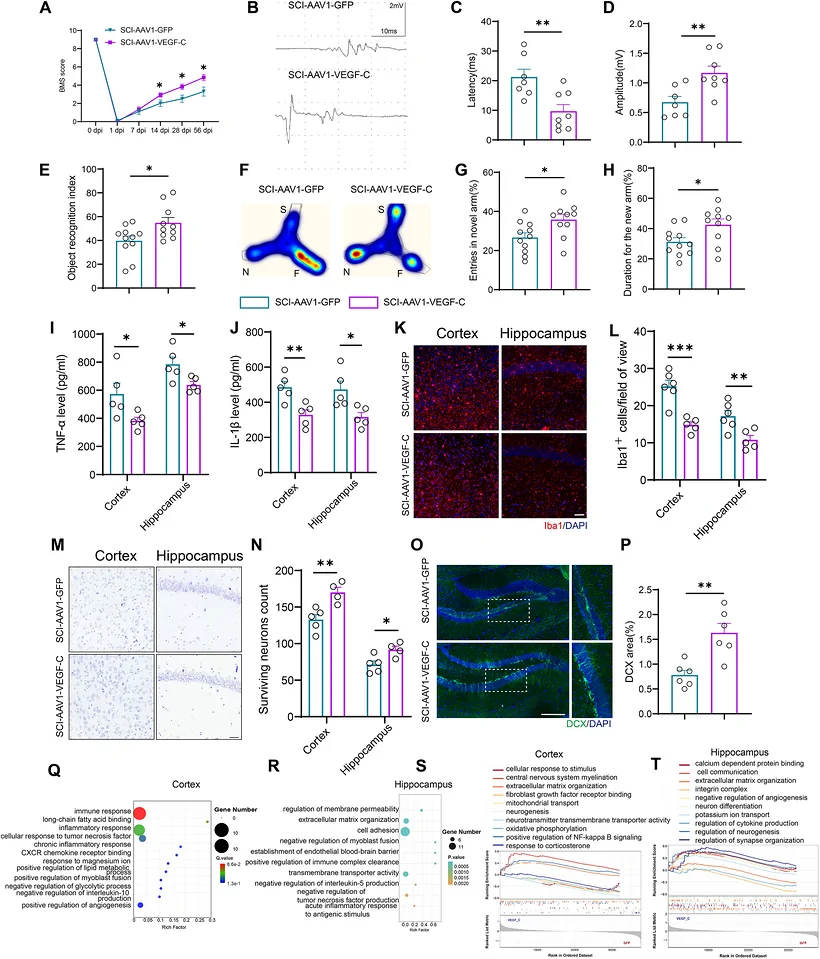
The effect of VEGF‐C treatment on secondary brain pathology after SCI. (A) BMS scores in SCI mice injected with AAV1‐GFP or AAV1‐VEGF‐C from 0 to 56 days post‐injury (dpi) (n = 10‐11mice per group). Statistical significance was determined by repeated measures ANOVA. (B) Representative MEP traces from SCI mice injected with AAV1‐GFP or AAV1‐VEGF‐C at 55 dpi. (C, D) Quantification of MEP latency and amplitude in SCI mice injected with AAV1‐GFP or AAV1‐VEGF‐C at 56 dpi (n = 10‐11 mice per group). (E) Object recognition index of NOR test in SCI mice injected with AAV1‐GFP or AAV1‐VEGF‐C at 56 dpi (n = 10‐11 mice per group). (F) Representative Y‐maze heatmaps showing exploration patterns of SCI mice injected with AAV1‐GFP or AAV1‐VEGF‐C at 54 dpi (S: start arm, N: novel arm, F: familiar arm). (G, H) Quantification of the number of entries into the novel arm and the duration spent in the novel arm in SCI mice injected with AAV1‐GFP or AAV1‐VEGF‐C at 56 dpi (n = 10‐11 mice per group). (I, J) ELISA analysis of IL‐1β and TNF‐α in the cortex and hippocampus of SCI mice injected with AAV1‐GFP or AAV1‐VEGF‐C at 56 dpi (n = 5 mice per group). (K, L) Representative immunofluorescence images and quantification of Iba1^+^ microglia (red) and DAPI(blue) in the cortex and hippocampus of SCI mice injected with AAV1‐GFP or AAV1‐VEGF‐C at 56 dpi (n = 5‐6 mice per group). Scale bar, 50 µm. (M, N) Representative Nissl staining images and quantification of surviving neurons in the cortex and hippocampus of SCI mice injected with AAV1‐GFP or AAV1‐VEGF‐C at 56 dpi (n = 4‐5 mice per group). Scale bar, 50 µm. (O, P) Representative immunofluorescence images and quantification of DCX^+^ neuronal progenitors (green) and DAPI (blue) in the dentate gyrus of SCI mice injected with AAV1‐GFP or AAV1‐VEGF‐C at 56 dpi (n = 6 mice per group). Scale bar, 200 µm. (Q, R) GO enrichment analysis of differentially expressed genes (DEGs) in the cortex and hippocampus of SCI mice injected with AAV1‐VEGF‐C compared to AAV1‐GFP controls at 56 dpi (n = 4 mice per group). (S, T) Enrichment plots from GSEA in the cortex and hippocampus of SCI mice injected with AAV1‐VEGF‐C versus AAV1‐GFP at 56 dpi. Statistical significance was determined by unpaired two‐tailed Student's t‐test. Data are presented as mean ± SEM. ^*^ *p* < 0.05, ^**^ *p* < 0.01, ^***^ *p* < 0.001.

We then examined whether restoring mLVs function confers neurobehavioral benefits post‐SCI. AAV1‐VEGF‐C treated mice achieved higher BMS scores from 14 dpi onward (Figure 3A). MEP recordings at 56 dpi further showed shorter latencies and larger amplitudes than in AAV1‐GFP treated SCI controls (Figure 3B–D). In cognitive testing, AAV1‐VEGF‐C treatment improved performance in both the NOR and Y‐maze tasks (Figure 3E–H).

Pathological assessments were conducted to elucidate the underlying mechanisms. VEGF‐C overexpression significantly reduced the levels of pro‐inflammatory cytokines in both the cortex and hippocampus (Figure 3I,J), decreased Iba1‐positive microglia (Figure 3K,L), increased neuronal survival (Figure 3M,N), and restored dentate gyrus neurogenesis (Figure 3O,P). RNA‐seq of cortical and hippocampal tissue showed that VEGF‐C overexpression modulated gene programs related to immune responses and neurogenesis (Figure 3Q,R). GSEA further confirmed significant enrichment of genes associated with cytokine production and neuroinflammatory processes (Figure 3S,T). Collectively, these findings demonstrate that therapeutic restoration of meningeal lymphatic function reduces secondary brain injury and improves motor and cognitive recovery after SCI.

### 2.5 LTBR Downregulation in Meningeal LECs Mediates Meningeal Lymphatic Dysfunction After SCI

To elucidate the cellular and molecular mechanisms underlying SCI‐induced meningeal lymphatic damage, we performed single‐cell RNA sequencing (scRNA‐seq) on the dura mater from sham and SCI mice at 56 dpi (Figure 4A). Quality control assessment showed comparable distributions of mitochondrial transcript percentages (Percent_mt) and total RNA counts (nCount_RNA) across individual samples, supporting consistent cellular quality and sequencing depth across the dataset (Figure S5A,B). Unsupervised clustering analysis identified 14 distinct cell clusters, with no overt segregation observed between the SCI and sham groups (Figure 4B). Each cluster was annotated based on established canonical markers, revealing the following major cell types (Figure 4D): neurons (*Cntnap2, Id4, Pax6*), oligodendrocytes (*Plp1, Mbp, Mpz*), ciliated ependymal cells (CEPECs; *Clic6, Enpp2, Kl*), neuroendocrine cells (*Chga, Chgb, Meg3*), endothelial cells (*Pecam1, Cdh5, Cldn5*), fibroblasts (*Dcn, Col1a1, Col1a2*), mural cells (*Acta2, Rgs5, Tagln*), B cells (*Ms4a1, Cd79a, Cd19*), T cells (*Cd3d, Trac, Trbc2*), natural killer (NK) cells (*Klrc1, Kird1, Xcl1*), mononuclear phagocytes (MPs; *Cd163, C1qc, Cd68*), plasmacytoid dendritic cells (pDCs; *Siglech, Ccr9, Irf8*), mast cells (*Tpsab1, Cma1, Cpa3*), and neutrophils (*S100a8, S100a9, Csf3r*). Although all 14 cell types were present in both the sham and SCI groups, their relative proportions exhibited distinct dynamic changes. Notably, endothelial cells constituted the most abundant population and displayed the pronounced alteration (Figure 4C). Given that meningeal LECs originate from the endothelial lineage, we subclustered the endothelial compartment and identified four distinct subclusters (Figure 4E). This analysis revealed a reduced proportion of meningeal LECs in the SCI group compared to sham controls, consistent with mLVs loss observed histologically.

**FIGURE 4:**
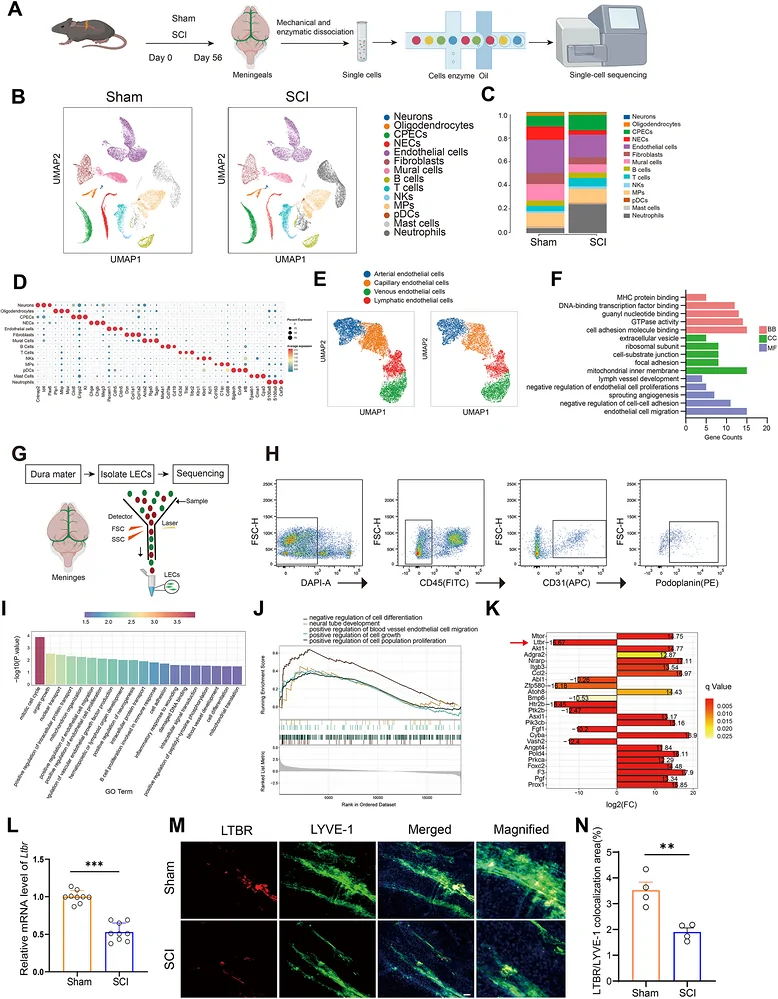
Transcriptomic changes in meningeal LECs after SCI implicate *Ltbr* as a key mediator of their dysfunction. (A) Experimental workflow and scRNA‐seq analysis of the dura mater from sham‐operated and SCI mice at 56 dpi. (B) Uniform manifold approximation and projection (UMAP) plots of all dural cells from sham and SCI groups, colored by cell type. (C) Bar chart comparing the proportion of each cell subcluster in sham versus SCI dura mater. (D) Dot plots depicting the average expression of canonical marker transcripts across 14 identified cell types. Dot size corresponds to the frequency of cells expressing each gene within the respective cell type. (E) Bar chart comparing the proportion of each endothelial cell subcluster in sham versus SCI dura mater. (F) Bar plot of GO enrichment analysis for LECs DEGs in sham versus SCI dura mater. n = 3 independent biological replicates per group with each replicate pooled from three mice. (G) Schematic of the strategy for isolating CD45^−^CD31^+^Podoplanin^+^ LECs from the dura mater for RNA‐seq analysis. (H) Representative dot and contour plots showing the gating strategy used to isolate meningeal LECs (DAPI^−^ CD45^−^ PDPN^+^ CD31^+^). (I, J) GO enrichment analysis and GSEA plots of DEGs in LECs from SCI mice compared to sham controls at 56 dpi. (K) Lollipop plot showing gene sets associated with proliferation, migration, and tube formation in LECs. n = 3 independent biological replicates per group with each replicate pooled from six mice. (L) qPCR analysis of *Ltbr* mRNA expression in the dura mater of sham and SCI mice at 56 dpi (n = 3 mice per group). (M, N) Representative dual‐immunofluorescence images and quantification of LTBR (red) and LYVE‐1 (green) co‐localization in the dura mater of sham and SCI mice at 56 dpi (n = 4 mice per group). Scale bar, 100 µm. Statistical significance was determined by unpaired two‐tailed Student's t‐test. Data are presented as mean ± SEM. ^**^ *p* < 0.01, ^***^ *p* < 0.001.

The LECs population was annotated based on the combined expression of lymphatic endothelial‐associated markers, including *Flt4*, *Lyve1*, and *Prox1* (Figure S5C). To elucidate the transcriptional alterations in LECs, we conducted GO enrichment analysis on LECs from both groups. The results indicated that LECs from SCI mice exhibited transcriptional changes predominantly associated with biological processes such as cell proliferation, migration, and tube formation (Figure 4F). However, due to the limited cell yield from dural scRNA‐seq and inherent technical constraints, we sought to validate and extend these findings. To this end, we isolated meningeal LECs from an additional cohort of SCI and sham mice using fluorescence‐activated cell sorting (FACS) based on a predefined gating strategy, followed by low‐input bulk RNA‐seq (Figure 4G,H). In agreement with the scRNA‐seq data, GO and GSEA analyses showed that SCI primarily affected pathways involved in cell proliferation, adhesion, migration, and lymphatic development (Figure 4I,J). Unexpectedly, pathway enrichment analysis revealed that the differentially expressed genes in LECs following SCI were not significantly associated with the classical VEGF‐C signaling pathway (Figure S6A–E). Subsequent examination of specific mRNAs implicated in these processes revealed decreased expression of multiple genes previously reported to promote LECs proliferation, migration, and tubulogenesis. Among the differentially expressed genes, we identified *Ltbr* as a candidate mediator of SCI‐induced lymphatic impairment (Figure 4K). qPCR confirmed significant downregulation of *Ltbr* transcription in the dura mater after SCI, consistent with our transcriptomic data (Figure 4L). This finding was further validated at the protein level using double immunofluorescence staining, which demonstrated a marked reduction of LTBR protein in LYVE‐1‐positive meningeal LECs following SCI (Figure 4M,N). These results identify LTBR downregulation in meningeal LECs as a potential mechanism of impaired lymphatic repair after SCI.

To further characterize the cellular context of meningeal LECs within the dural microenvironment, we performed cell‐cell communication analysis, which revealed predicted interactions between LECs and multiple stromal and immune cell populations, with fibroblasts showing the strongest overall communication (Figure S5D).

### 2.6 *Ltbr* Knockdown Exacerbates Meningeal Lymphatic Impairment After SCI

To determine whether Ltbr is required for maintaining basal meningeal lymphatic function under physiological conditions, we examined the effects of *Ltbr* deficiency in wild‐type mice in the absence of SCI. Mice received intracisternal administration of AAV9‐shLtbr or control AAV9‐nc, and meningeal lymphatic drainage was evaluated 28 days later. Compared with control mice, *Ltbr*‐deficient mice exhibited no significant changes in OVA‐647 accumulation in the dCLNs (Figure S7A,B) and LYVE‐1‐positive vessel area fraction in dura mater (Figure S7C,D), indicating that LTBR deficiency alone does not compromise resting meningeal lymphatic drainage. To further determine whether LTBR contributes to lymphatic repair following SCI, we next examined the effects of *Ltbr* knockdown in the SCI model (Figure 5A). Successful *Ltbr* mRNA knockdown in meningeal LECs was confirmed before SCI (Figure 5B). At 56 dpi, mice with *Ltbr* knockdown showed less OVA‐647 accumulation in the dCLNs than control mice, indicating further impairment of lymphatic drainage (Figure 5C–F). Consistently, LYVE‐1 immunofluorescence revealed a greater reduction in mLVs coverage (Figure 5G,H), and tracer influx into the brain parenchyma was further diminished (Figure 5I,J), reflecting aggravated glymphatic dysfunction.

**FIGURE 5:**
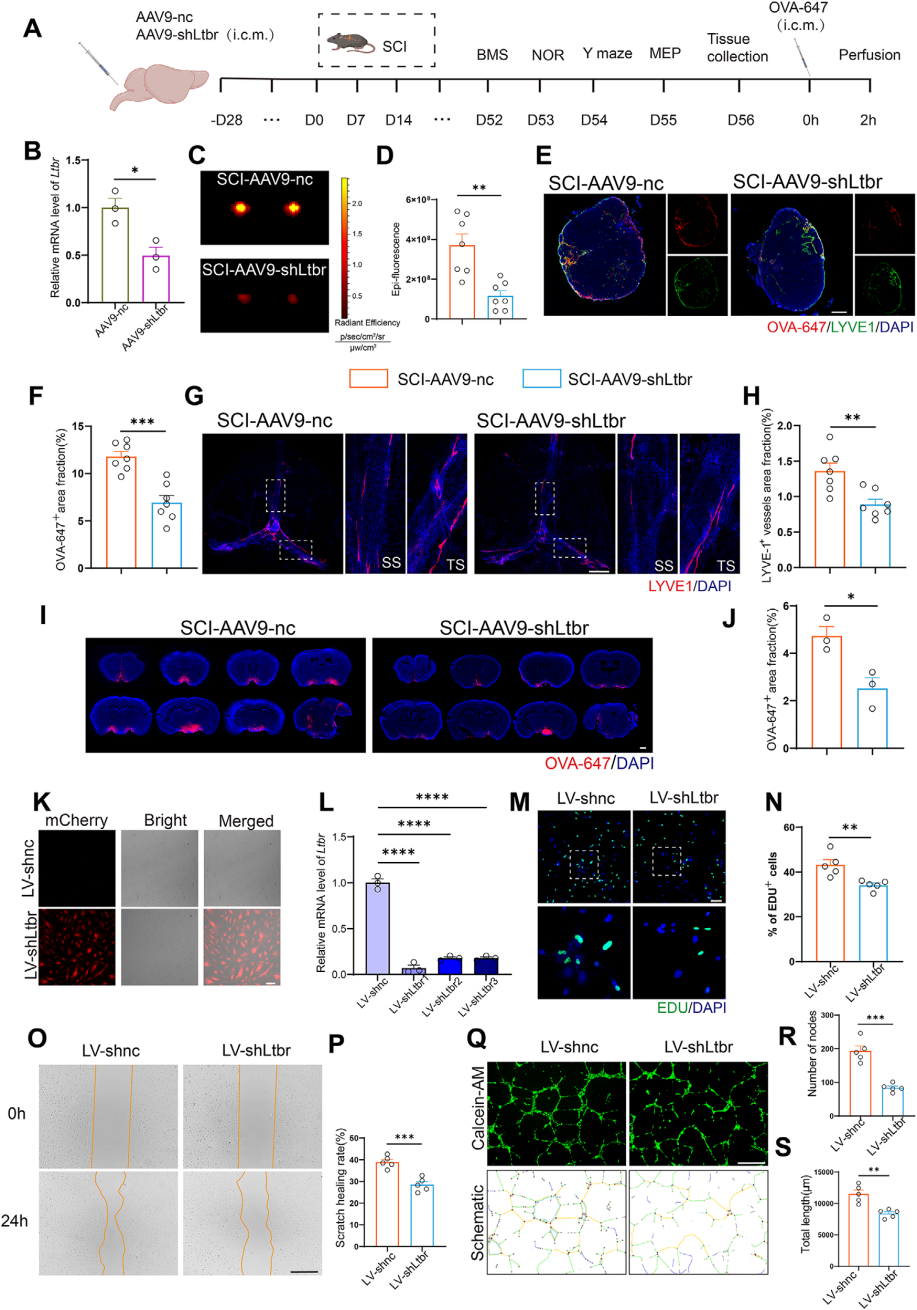
The effect of *Ltbr* knockdown on meningeal lymphatic function after SCI. (A) Schematic timeline of experimental procedures. (B) Validation of efficient *Ltbr* knockdown in mouse meninges after AAV9‐shLtbr injection, assessed by qPCR (n = 3 mice per group). (C, D) Representative in vivo fluorescence images and quantification of OVA‐647 tracer drainage to dCLNs at 2 h after i.c.m. injection in SCI‐AAV9‐nc and SCI‐AAV9‐shLtbr mice (n = 7 mice per group). (E, F) Representative immunofluorescence images and quantification of dCLNs sections stained for OVA‐647 (red), LYVE‐1 (green), and DAPI (blue) at 56 dpi in SCI‐AAV9‐nc and SCI‐AAV9‐shLtbr mice (n = 6 mice per group), scale bar, 200 µm. (G, H) Representative immunofluorescence images (LYVE‐1, red; DAPI, blue) and quantification of LYVE‐1 coverage in SCI‐AAV9‐nc and SCI‐AAV9‐shLtbr mice (n = 6 mice per group). scale bar, 1000 µm. (I, J) Representative images and quantification of OVA‐647 tracer perfusion into the brain parenchyma in SCI‐AAV9‐nc and SCI‐AAV9‐shLtbr mice (n = 3 mice per group), scale bar, 1000 mm. Statistical significance was determined using an unpaired two‐tailed Student's t‐test. (K, L) Representative images of mCherry fluorescence confirming successful lentiviral transduction of LV‐shnc or LV‐shLtbr in hLECs, scale bar, 100 µm, and validation of *Ltbr* mRNA knockdown by qPCR (n = 3 independent biological replicates). Statistical analysis was performed by one‐way ANOVA followed by Dunnett's multiple comparisons test. (M, N) Representative immunofluorescence images (EdU, green; DAPI, blue) and quantitative analysis of EdU‐positive proliferating LECs in the LV‐shnc and LV‐shLtbr groups (n = 5 independent biological replicates), scale bar, 100 µm. (O, P) Representative images and quantification of scratch wound healing assays in LV‐shnc and LV‐shLtbr LECs at 0 and 24 h (n = 5 independent biological replicates), scale bar, 200 µm. (Q–S) Representative images and quantification of tube formation assays in LV‐shnc and LV‐shLtbr LECs, showing the number of nodes and total length (n = 5 independent biological replicates), scale bar, 200 µm. Statistical significance was determined using an unpaired two‐tailed Student's t‐test. Data are presented as mean ± SEM. ^*^ *p* < 0.05, ^**^ *p* < 0.01, ^***^ *p* < 0.001, ^****^ *p* < 0.0001.

To assess the effects of LTBR on LECs function, we performed lentivirus‐mediated *Ltbr* silencing in human lymphatic endothelial cells (hLECs) (Figure 5K,L). *Ltbr* silencing significantly reduced the EdU‐positive cell rate (Figure 5M,N), decreased the wound closure rate in scratch assays (Figure 5O,P), and diminished the number of nodes and total length in tube formation assays (Figure 5Q–S). These results indicate that LTBR is required for LECs proliferation, migration, and tube formation, providing a cellular explanation for the in vivo lymphatic deficits.

### 2.7 *Ltbr* Knockdown Aggravates Neurological Deficits and Secondary Brain Pathology After SCI

We next determined whether *Ltbr* knockdown in meningeal LECs exacerbates secondary brain pathology following SCI. Notably, *Ltbr*‐knockdown SCI mice exhibited significantly lower BMS scores compared to control SCI mice from 14 through 56 dpi (Figure 6A), indicating that *Ltbr* suppression impairs locomotor recovery. MEP recordings at 56 dpi revealed that *Ltbr* knockdown further increased latency and reduced amplitude (Figure 6B–D), suggesting exacerbated corticospinal conduction dysfunction. In cognitive tests, *Ltbr*‐knockdown mice showed reduced preference for the novel object in the NOR task and impaired performance in the Y‐maze (Figure 6E–H).

**FIGURE 6:**
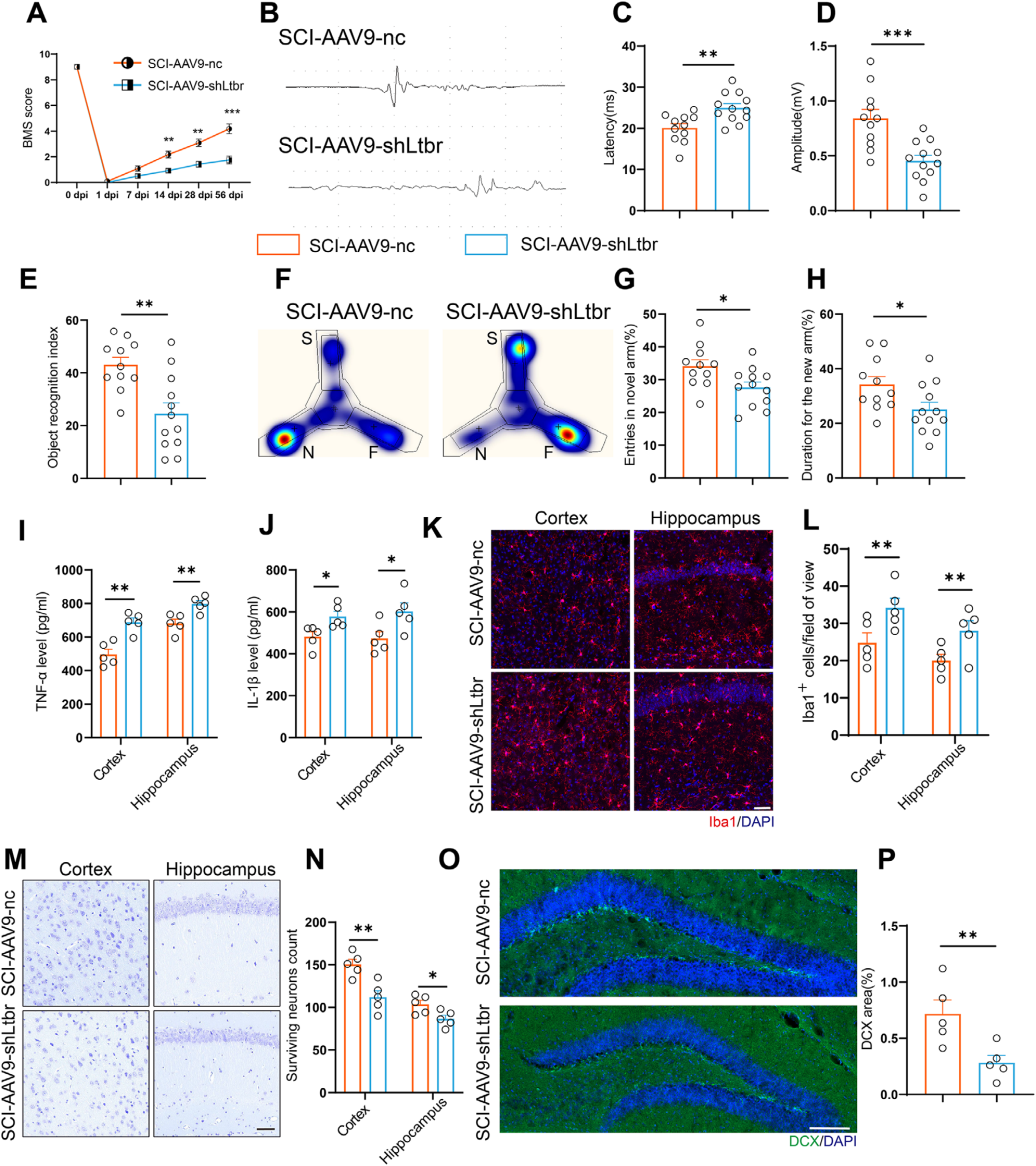
The effect of *Ltbr* knockdown on neurological deficits and secondary brain pathology after SCI. (A) Time course of BMS scores evaluating locomotor function in SCI‐AAV9‐nc and SCI‐AAV9‐shLtbr mice from 0 to 56 dpi (n = 11–12 mice per group). Statistical significance was determined by repeated measures ANOVA. (B–D) Representative MEP traces and quantification of MEP latency and amplitude in SCI‐AAV9‐nc and SCI‐AAV9‐shLtbr mice (n = 11–12 mice per group). (E) Quantification of the object recognition index in the NOR test in SCI‐AAV9‐nc and SCI‐AAV9‐shLtbr mice (n = 11–12 mice per group). (F–H) Representative Y‐maze heatmaps and quantification of entries into the novel arm and duration spent in the novel arm in SCI‐AAV9‐nc and SCI‐AAV9‐shLtbr mice (n = 11–12 mice per group). (I, J) ELISA quantification of TNF‐α and IL‐1β levels in the cortex and hippocampus of SCI‐AAV9‐nc and SCI‐AAV9‐shLtbr mice (n = 5 mice per group). (K, L) Representative immunofluorescence images and quantification of Iba1‐positive microglia (red) and DAPI (blue) in the cortex and hippocampus in SCI‐AAV9‐nc and SCI‐AAV9‐shLtbr mice (n = 5 mice per group), scale bar, 50 µm. (M, N) Representative Nissl staining images and quantification of surviving neurons in the cortex and hippocampus in SCI‐AAV9‐nc and SCI‐AAV9‐shLtbr mice (n = 5 mice per group), scale bar, 50 µm. (O, P) Representative immunofluorescence images and quantification of DCX‐positive newborn neurons (green) and DAPI (blue) in the hippocampal dentate gyrus in SCI‐AAV9‐nc and SCI‐AAV9‐shLtbr mice (n = 5 mice per group), scale bar, 200 µm. Statistical significance was determined using an unpaired two‐tailed Student's t‐test. Data are presented as mean ± SEM. ^*^ *p* < 0.05, ^**^ *p* <0.01, ^***^ *p* < 0.001, ^****^ *p* < 0.0001.

Consistent with behavioral deficits, *Ltbr* knockdown intensified SCI‐induced secondary brain pathology. The levels of pro‐inflammatory cytokines TNF‐α and IL‐1β were further increased in both the cortex and hippocampus (Figure 6I,J), and Iba1‐positive microglia accumulation was enhanced (Figure 6K,L), indicating exacerbated neuroinflammation. Neuronal loss in the cortex and hippocampus was more severe, and dentate gyrus neurogenesis was further reduced following Ltbr knockdown (Figure 6M–P). Collectively, these results demonstrate that LTBR downregulation in meningeal LECs exacerbates SCI‐induced meningeal lymphatic dysfunction, neuroinflammation, neuronal injury, and neurological deficits.

### 2.8 *Ltbr* Overexpression Promotes Meningeal Lymphatic Restoration With Potential Involvement of PI3K‐AKT Signaling After SCI

To investigate whether enhancing LTBR expression is sufficient to promote meningeal lymphatic restoration after SCI, we generated mice with inducible *Ltbr* overexpression in LECs by crossing Prox1‐CreERT2 mice with conditional *Ltbr* knock‐in mice with *Ltbr* ^flox/flox^ mice (Figure 7A, Figure S8A). Following intraperitoneal tamoxifen administration, we validated significant upregulation of LTBR levels specifically in the meningeal LECs of Ltbr^CKI/+^; Prox1‐Cre mice, compared with Ltbr^CKI/+^ littermate controls (Figure S8B,C). Remarkably, Ltbr overexpression significantly increased OVA‐647 drainage to the dCLNs, expanded LYVE‐1‐positive coverage area of mLVs, and enhanced tracer influx into the brain parenchyma following SCI (Figure 7B–I), indicating improved meningeal lymphatic and glymphatic function.

**FIGURE 7:**
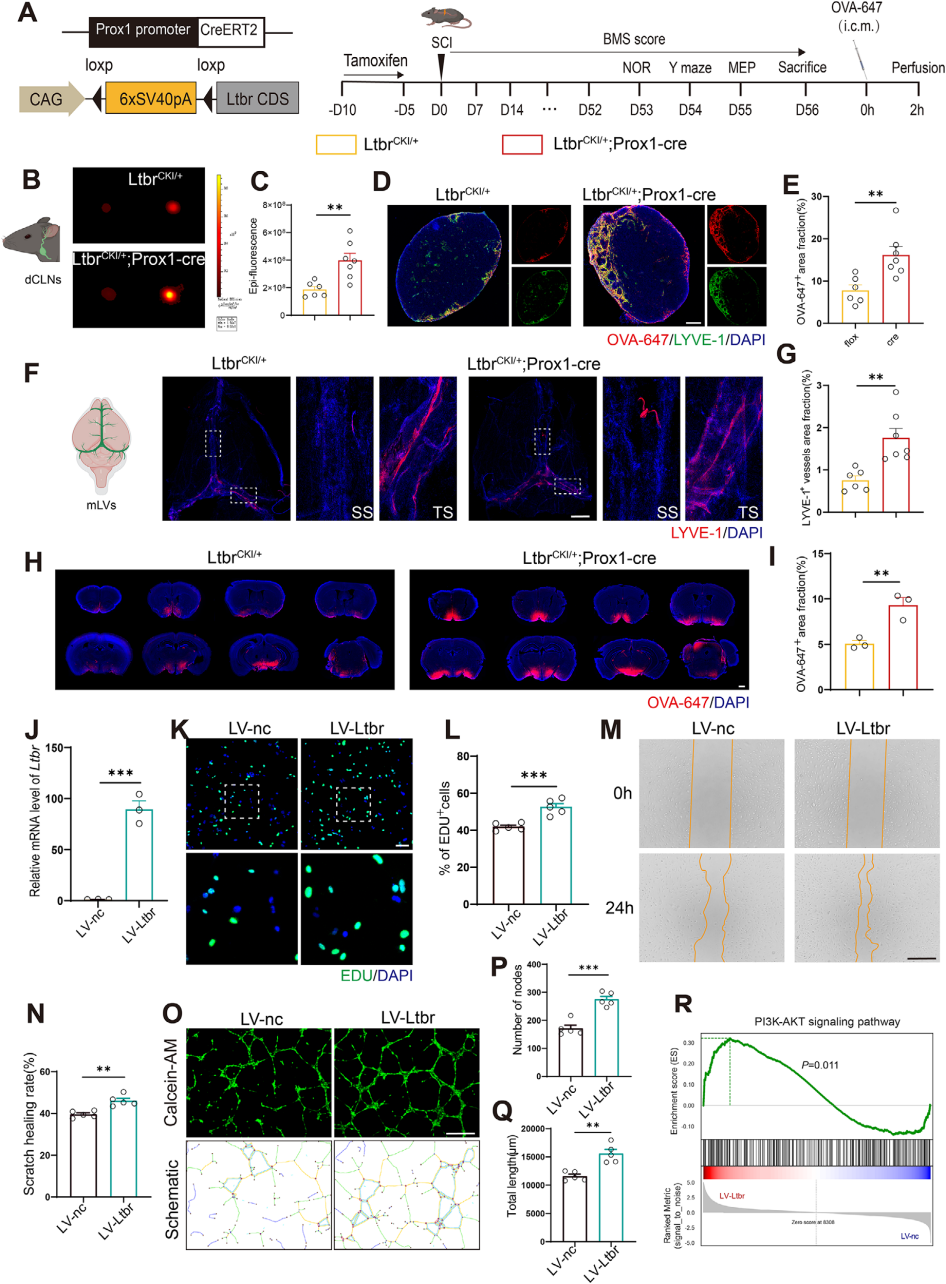
The effect of *Ltbr* overexpression on meningeal lymphatic restoration and PI3K‐AKT signaling after SCI. (A) Schematic diagram illustrating the generation strategy of Ltbr^CKI/+^; Prox1‐Cre mice and the subsequent experimental design. These mice enable tamoxifen‐inducible overexpression of *Ltbr* specifically in LECs. (B, C) Representative in vivo imaging and quantitative analysis of OVA‐647 tracer intensity in the dCLNs between two groups of mice after SCI (n = 6–7 mice per group). (D, E) Representative images and quantification of OVA‐647 tracer drainage to the dCLNs (n = 6 mice per group), scale bar, 100 µm. (F, G) Representative immunofluorescence images of LYVE‐1(red) and DAPI (blue) in the dura mater between two groups of mice after SCI, and quantification of LYVE‐1^+^ coverage area (n = 6 mice per group), scale bar, 100 µm. (H, I) Representative images showing the distribution of i.c.m‐injected OVA‐647 tracer (red) and DAPI staining (blue) in brain sections, and quantification of the distribution of tracer in multiple brain regions (n = 3 mice per group), scale bar, 1000 mm. (J) Validation of *Ltbr* mRNA overexpression in hLECs transduced with LV‐Ltbr lentivirus, compared to the LV‐nc group (n = 3 independent biological replicates). (K, L) Representative images and quantification of EdU staining (green) and DAPI(blue) in hLECs transduced with LV‐nc or LV‐Ltbr lentivirus (n = 5 independent biological replicates), scale bar, 100 µm. (M, N) Representative images and quantification of scratch wound healing assays in LV‐nc and LV‐Ltbr LECs at 0 and 24 h (n = 5 independent biological replicates), scale bar, 200 µm. (O–Q) Representative images of tube formation assays and quantitative analysis of node number and total tube length in LV‐nc and LV‐Ltbr LECs (n = 5 independent biological replicates), scale bar, 200 µm. (R) GSEA enrichment plots based on *Ltbr* overexpression in hLECs (n = 4–5 independent biological replicates). Statistical significance was determined using an unpaired two‐tailed Student's t‐test. Data are presented as mean ± SEM. ^*^ *p* < 0.05, ^**^ *p* < 0.01, ^***^ *p* < 0.001, ^****^ *p* < 0.0001.

To explore the cellular mechanisms by which LTBR regulates LECs function, we performed in vitro assays using hLECs. Lentiviral‐mediated overexpression of Ltbr (LV‐Ltbr) significantly increased cell proliferation, accelerated migration, and enhanced tube formation compared with the control virus (Figure 7J–Q), demonstrating that LTBR is sufficient to promote core lymphangiogenic behaviors. To define the molecular mechanisms of these effects, we performed bulk RNA‐seq in hLECs transfected with LV‐Ltbr or vector. GSEA revealed significant enrichment of the PI3K‐AKT signaling pathway following *Ltbr*‐overexpression (Figure 7R). To further validate this association, we performed pharmacological inhibition using GDC‐0941. A dose‐response analysis yielded an IC50 of 1.423 µm(Figure S9A), and 0.5 µm GDC‐0941 was selected for subsequent experiments. GDC‐0941 attenuated LTBR‐associated PI3K‐AKT activation and partially reversed the LTBR‐induced enhancement of LECs proliferation, migration, and tube formation (Figure S9B–J). Given the established role of PI3K‐AKT signaling in regulating LECs proliferation, migration, and morphogenesis [21], these findings suggest that PI3K‐AKT signaling is involved in LTBR‐mediated regulation of LECs functions.

### 2.9 *Ltbr* Overexpression Improves Neurological Function Recovery and Ameliorates Secondary Brain Pathology After SCI

Finally, we tested whether LTBR‐driven lymphatic repair translates into improved neurological outcomes after SCI. Locomotor function evaluation showed *Ltbr*‐overexpressing mice exhibited significantly higher BMS scores compared with littermate controls from 14 dpi onward (Figure 8A). MEP recordings at 56 dpi revealed decreased latency and increased amplitude in *Ltbr*‐overexpressing SCI mice (Figure 8B–D), indicating enhanced corticospinal tract conduction. *Ltbr*‐overexpressing mice also improved performance in the NOR and Y‐maze tests (Figure 8E–H), indicating enhanced cognitive recovery. Neuropathological examination at 56 dpi revealed that *Ltbr* overexpression attenuated SCI‐induced brain pathology. The levels of pro‐inflammatory cytokines and the density of Iba1‐positive microglia were significantly reduced in both the cortex and hippocampus of *Ltbr*‐overexpressing mice, indicating mitigated neuroinflammation (Figure 8I–L). Neuronal survival was preserved in the cortex and hippocampus, and hippocampal neurogenesis was increased following *Ltbr* overexpression (Figure 8M–P). Together, these results demonstrate that *Ltbr* overexpression in meningeal LECs restores meningeal lymphatic structure and function, thus attenuating secondary brain neuroinflammation and neuronal damage, and significantly improving both motor and cognitive recovery after SCI.

**FIGURE 8:**
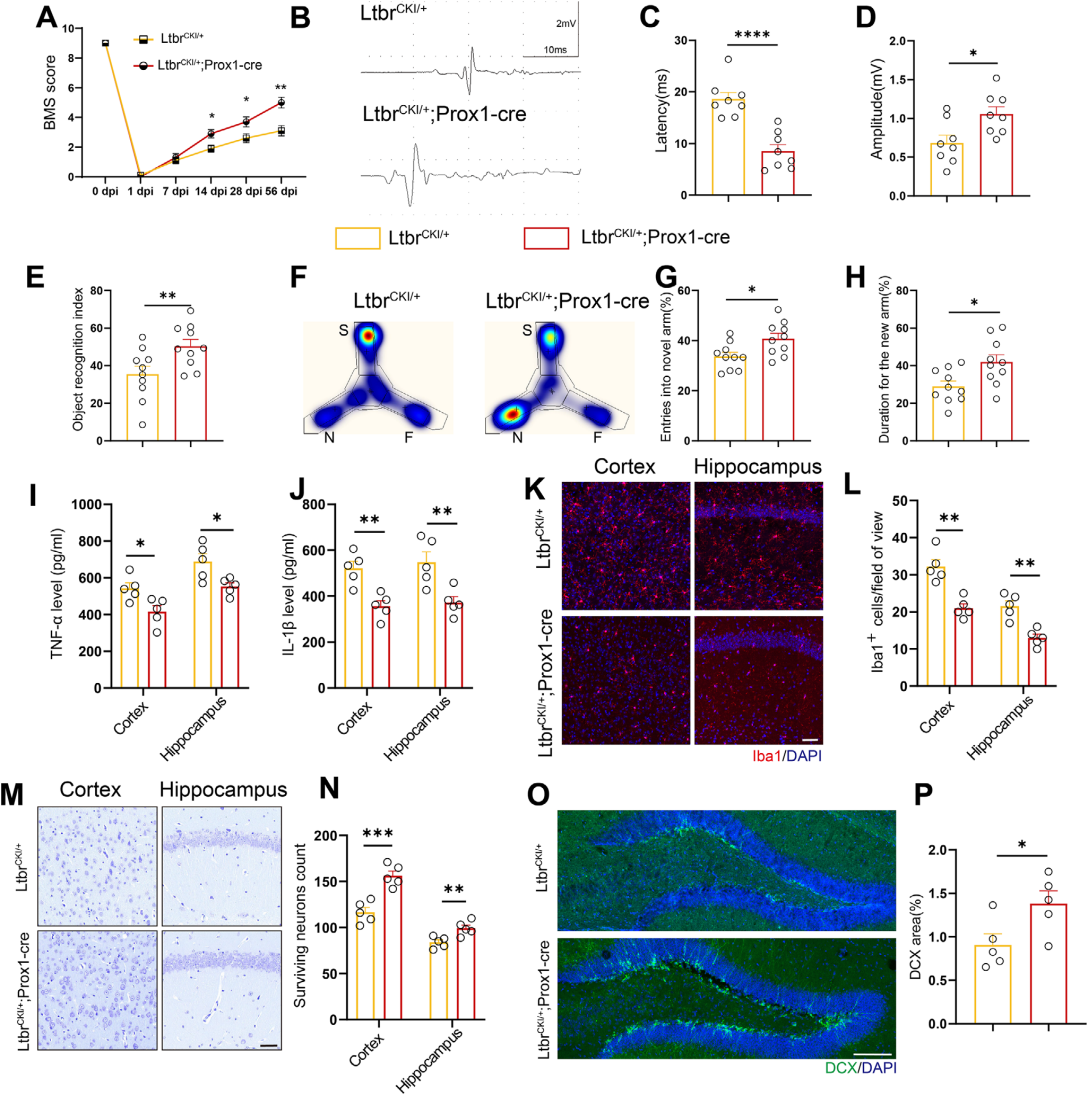
The effect of *Ltbr* overexpression on neurological recovery and secondary brain pathology after SCI. (A) BMS scores in Ltbr^CKI/+^ and Ltbr^CKI/+^; Prox1‐Cre mice from 0 to 56 days post‐injury (dpi) (n = 10 mice per group). (B) Representative MEP traces from Ltbr^CKI/+^ and Ltbr^CKI/+^; Prox1‐Cre mice at 56 dpi. Statistical significance was determined by repeated measures ANOVA. (C, D) Quantification of MEP latency and amplitude in Ltbr^CKI/+^ and Ltbr^CKI/+^; Prox1‐Cre mice at 56 dpi (n = 8 mice per group). (E) Quantification of the object recognition index in the NOR test between two groups after SCI (n = 10 mice per group). (F) Representative Y‐maze heatmaps showing exploration patterns of Ltbr^CKI/+^ and Ltbr^CKI/+^; Prox1‐Cre mice at 56 dpi after SCI (S: start arm, N: novel arm, F: familiar arm). (G, H) Quantification of the number of entries into the novel arm and the duration spent in the novel arm in Ltbr^CKI/+^ and Ltbr^CKI/+^; Prox1‐Cre mice after SCI (n = 10 mice per group). (I, J) TNF‐α and IL‐1β levels in the cortex and hippocampus of Ltbr^CKI/+^ and Ltbr^CKI/+^; Prox1‐Cre mice at 56 dpi (n = 5 mice per group). (K, L) Representative immunofluorescence images(microglia, red; DAPI, blue) and quantification of Iba1^+^microglia in the cortex and hippocampus of Ltbr^CKI/+^ and Ltbr^CKI/+^; Prox1‐Cre mice at 56 dpi (n = 5 mice per group). Scale bar, 50 µm. (M, N) Representative Nissl staining images and quantification of surviving neurons in the cortex and hippocampus of Ltbr^CKI/+^ and Ltbr^CKI/+^; Prox1‐Cre mice at 56 dpi (n = 5 mice per group). Scale bar, 50 µm. (O, P) Representative immunofluorescence images and quantification of DCX^+^ neuronal progenitors (green) and DAPI (blue) in the hippocampal dentate gyrus of Ltbr^CKI/+^ and Ltbr^CKI/+^; Prox1‐Cre mice at 56 dpi (n = 5 mice per group). Scale bar, 200 µm. Statistical significance was determined using an unpaired two‐tailed Student's t‐test. Data are presented as mean ± SEM. ^*^ *p* < 0.05, ^**^ *p* < 0.01, ^***^ *p* < 0.001.

## 3 Discussion

SCI is increasingly recognized as a CNS‐wide disorder rather than a lesion confined to the injured spinal cord. Our study identifies meningeal lymphatic dysfunction as an important contributor to remote secondary brain pathology after SCI. Across human imaging, mouse models, and molecular gain‐ and loss‐of‐function approaches, we found that SCI impaired meningeal lymphatic structure and function, reduced CSF drainage and glymphatic perfusion, and was associated with neuroinflammation, neuronal loss, impaired hippocampal neurogenesis, and motor and cognitive deficits. Integrated single‐cell and bulk transcriptomic analyses further identified reduced LTBR expression in meningeal LECs as a molecular change related to impaired lymphatic repair after SCI. Together, these findings extend the concept of secondary brain pathology after SCI to the meningeal lymphatic system.

A growing body of work has shown that SCI induces secondary structural and functional alterations in remote brain regions [22, 23], particularly the motor cortex and hippocampus [24, 25], and that supraspinal neuroinflammation, neuronal injury, and altered inter‐organ signaling may contribute to these abnormalities [1, 26]. Another line of evidence suggests that extracellular vesicles from the injured spinal cord can enter the blood and trigger inflammatory and degenerative responses in the brain [27]. These findings suggest that disability after SCI cannot be attributed solely to local spinal damage. Instead, complex crosstalk between the spinal cord and remote brain regions contributes significantly. Our findings add meningeal lymphatic dysfunction to this complex network of pathological responses. This interpretation is also consistent with studies demonstrating roles for meningeal lymphatics in CSF drainage, solute clearance, and immune surveillance [15], as well as evidence linking lymphatic dysfunction to impaired fluid clearance and neuroinflammation in traumatic brain injury, neurodegenerative disease, and CNS inflammation [28, 29, 30]. Our results therefore extend these observations to SCI and suggest that impaired meningeal lymphatic function may represent an additional pathway linking spinal injury to remote brain pathology.

The human imaging findings provide clinical evidence of altered glymphatic‐related function after SCI. Patients with SCI had a lower ALPS index than healthy controls, suggesting impaired glymphatic function, and also exhibited cortical and hippocampal atrophy. The observed association between a lower ALPS index and reduced brain volume suggests that glymphatic‐related abnormalities are associated with structural brain alterations after SCI. However, DTI‐ALPS is an indirect measure and does not directly assess meningeal lymphatic outflow [31]. Therefore, the clinical data cannot determine whether meningeal lymphatic dysfunction accompanies or contributes to the observed glymphatic‐related abnormalities in patients with SCI. In our mouse models, meningeal lymphatic drainage and glymphatic‐related tracer transport were evaluated separately, allowing us to demonstrate that both processes are impaired after SCI. Nevertheless, whether this relationship is recapitulated in patients with SCI remains to be determined.

Our molecular analyses further identify LTBR as a potential regulator of meningeal lymphatic repair after SCI. Notably, AAV‐mediated *Ltbr* knockdown in wild‐type mice under physiological conditions did not significantly alter basal meningeal lymphatic drainage, suggesting that LTBR is not essential for maintaining resting lymphatic function. In contrast, *Ltbr* knockdown aggravated meningeal lymphatic structural and functional impairment and secondary brain pathology after SCI, whereas *Ltbr* overexpression promoted lymphatic repair and improved neurological outcomes. This aligns with previous reports showing that LTBR signaling supports lymphatic endothelial proliferation, tube formation, and lymphoid stromal organization in peripheral tissues [32, 33, 34]. In cultured hLECs, LTBR enhanced proliferation, migration, and tube formation, supporting a cell‐intrinsic role in lymphatic remodeling. Together, these findings suggest that LTBR may play a more prominent role in injury‐induced lymphatic adaptation and repair rather than in the maintenance of basal meningeal lymphatic function.

Transcriptomic analysis of LTBR‐overexpressing LECs implicated PI3K‐AKT signaling in LTBR‐associated cellular responses. Protein‐level validation and pharmacological PI3K inhibition further supported involvement of this pathway in LTBR‐mediated effects on LECs proliferation, migration, and tube formation. Given the established role of PI3K‐AKT signaling in endothelial survival and remodeling [35, 36], these findings suggest that PI3K‐AKT may contribute to LTBR‐mediated lymphatic repair. However, the present data do not establish PI3K‐AKT as the sole or obligatory downstream pathway, and in vivo genetic or pharmacological manipulation will be required to determine its contribution to LTBR‐dependent lymphatic repair after SCI.

These findings also have potential therapeutic implications, although important translational challenges remain. VEGF‐C promotes lymphangiogenesis and lymphatic expansion, whereas Piezo1 activation has been reported to enhance lymphatic function through mechanotransduction and increase CSF drainage [20, 37]. In our model, VEGF‐C improved meningeal lymphatic function and neurological outcomes, whereas LTBR appeared to promote the intrinsic reparative capacity of LECs. Thus, VEGF‐C, Piezo1, and LTBR may modulate complementary aspects of lymphatic restoration. However, our interventions were administered before SCI induction and therefore primarily establish preventive effects rather than therapeutic efficacy after established injury. Moreover, systemic LTBR manipulation may produce unintended immune effects because LTBR has established roles in lymphoid organogenesis and immune homeostasis [34]. The intracisternal AAV approach used here is also invasive and may limit clinical applicability. Selective and less invasive strategies for targeting meningeal LECs will therefore be required. The safety and efficacy of small‐molecule LTBR agonists for meningeal lymphatic applications have yet to be fully established [38], making pharmacological development an important future direction rather than an immediately translatable strategy.

Our study has several limitations. The human data are cross‐sectional and therefore cannot establish temporal or causal relationships between glymphatic dysfunction and brain atrophy after SCI. Moreover, DTI‐ALPS provides an indirect measure of perivascular fluid transport and does not directly assess meningeal lymphatic drainage. Secondary brain injury after SCI is a multifactorial process involving neural, immune, vascular, endocrine, and humoral mechanisms [5]; thus, meningeal lymphatic dysfunction is unlikely to be the sole contributor. Although LTBR emerged as an important regulator of lymphatic repair, the upstream signals responsible for its downregulation after SCI remain unknown. Systemic inflammatory mediators and circulating extracellular vesicles represent plausible candidates [39, 40], but whether they directly regulate *Ltbr* transcription requires further investigation. Finally, it is also unclear whether interactions between dural immune cells and LECs influence post‐injury lymphatic remodeling, whether sex differences exist, and whether LTBR‐targeted interventions can be delivered selectively and safely to meningeal LECs in clinically relevant settings.

## 4 Conclusion

In conclusion, these findings identify meningeal lymphatic dysfunction as an important component of secondary brain pathology after SCI and highlight LTBR as a potential regulator of lymphatic repair and a candidate target for future therapeutic investigation.

## 5 Materials and Methods

### 5.1 Participants and MRI Data Acquisition

This study was approved by the Ethics Committee of Army Medical University, Chongqing, China (Approval No.KY2020252), and written informed consent was obtained from all participants. A total of 72 participants were recruited, including 40 SCI patients (SP group) and 32 healthy controls (HC group) matched for age, sex, and educational level. MRI data were acquired using a Siemens Trio 3.0 T scanner. DTI was performed with 30 diffusion gradient directions (b = 1000 s/mm^2^), and T1‐weighted structural images were also acquired. The ALPS index was employed to evaluate glymphatic system function, following a previously described method [41]. All raw DTI data were preprocessed using FSL version 6.0 to generate fractional anisotropy (FA) maps and diffusivity maps along three orthogonal axes (Dxx: left‐right, Dyy: anterior‐posterior, Dzz: superior‐inferior). Two neuroimaging physicians, blinded to clinical information, independently placed circular regions of interest (ROI) with a diameter of 5 mm on the projection fibers (corticospinal tract) and association fibers (superior longitudinal fasciculus) at the level of the lateral ventricular body on the native‐space FA maps. Diffusivity values within each ROI were recorded, and the ALPS index was calculated using the following formula: ALPS=Mean(Dx_proj,Dx_assoc)/Mean(Dy_proj,Dz_assoc). The ALPS index was calculated separately for the left and right hemispheres, and the bilateral average was used for subsequent analyses. High‐resolution T1‐weighted structural images were preprocessed using CAT12 (SPM12, version r1730) and FreeSurfer 7.0 software. Bilateral volumes of the precentral gyrus and hippocampus were extracted, and the sum of the left and right volumes was calculated. All volume values were normalized by total intracranial volume to account for individual differences in head size.

### 5.2 Animals

Female C57BL/6J mice aged 6–8 weeks were purchased from Hunan SJA Laboratory Animal Co., Ltd (Hunan, China). Prox1‐CreERT2 mice and LtbrCKI/+ mice (genetic background: C57BL/6J) were obtained from Cyagen Biosciences (Suzhou, China). The LtbrCKI/+ mice were mated with Prox1‐CreERT2 to generate LtbrCKI/+; Prox1‐Cre ERT2(LtbrCKI/+; Prox1‐Cre) mice in which conditional *Ltbr* knock‐in can be induced by tamoxifen administration. Littermate LtbrCKI/+ mice lacking Cre recombinase were used as controls. All animal experiments were approved by the Institutional Animal Care and Use Committee (AMUWEC20232650).

### 5.3 Mouse SCI Model Contusion

A contusive SCI was induced at the T9 vertebral level using a modified Allen's weight‐drop device as previously described [42]. Briefly, mice were anesthetized with tribromoethanol (20 µl/g, i.p.), and the T9 spinal cord was exposed following laminectomy. In sham‐operated animals, only laminectomy was performed. For injury groups, a moderate‐grade SCI was produced using an impact force of 70 dyn. Subsequently, the incisions were cleaned and sutured in layers. The bladders were manually voided twice daily until bladder function was restored. In the sham group, sham mice underwent the same procedure except for contusion. Animals with unsuccessful SCI induction were excluded.

### 5.4 Intra‐Cisterna Magna (i.c.m.) Injections of Drugs and Viral Vectors

Mice were anesthetized with tribromoethanol (20 µL/g) via intraperitoneal injection. The dorsal neck skin was shaved and disinfected with 70% ethanol, and the head was secured in a stereotaxic frame. A small incision was made at the base of the skull, and the muscle layers were separated to expose the cisterna magna. A 33‐gauge needle connected to a 10‐µL Hamilton microsyringe was used to carefully puncture the dura mater. All injections were performed using a microinjection pump (KDS LEGATO 130, RWD) at a rate of 1 µL/min. For tracer experiments, 10 µL of Alexa Fluor 647‐conjugated ovalbumin (OVA‐647, 2 mg/mL; Thermo Fisher Scientific) was injected. For AAV‐mediated gene delivery, 3 µL of virus solution was injected, including AAV1‐VEGF‐C (1.44 × 1012 genome copies/mL) and control AAV1‐GFP for VEGF‐C overexpression, as well as AAV9‐shLtbr (targeting mouse Ltbr, NM_010736.5; 8.77 × 1012 genome copies/mL) and control AAV9‐GFP (AAV9‐nc) for Ltbr knockdown (all viral vectors were obtained from Genechem Co., Ltd., Shanghai, China). Following each injection, the needle was left in place for an additional 5 min and then slowly withdrawn to prevent backflow. The wound was closed, and the mouse was placed on a heating pad for recovery.

### 5.5 mLVs Ablation

Visudyne was administered as previously described [43]. The compound was reconstituted according to the manufacturer's instructions(HY‐B0146, MedChemExpress) and injected into the cisterna magna at a rate of 2 µL/min for a total volume of 10 µL (2 mg/mL). Fifteen minutes post‐injection, photoconversion was performed by exposing five distinct cranial sites to non‐thermal 689 nm light: one at the injection site, one over the superior sagittal sinus, one at the confluence of sinuses (torcular), and two over the transverse sinuses. Each site received a light dose of 50 J/cm^2^ at an irradiance of 600 mW/cm^2^ for 83 s per spot. Control animals received an identical volume of Visudyne without subsequent light exposure. Following the procedure, the skin was sutured, and mice were placed on a heating pad for recovery.

### 5.6 Motor Function Assessment

Hindlimb motor function recovery was evaluated using the BMS. Mice were placed individually in an open field arena (dimensions: 62 × 42 cm) and observed for 5 min. Scoring was performed by two trained investigators who were blinded to the experimental group assignments of the animals. BMS scores were recorded pre‐injury and then at specified post‐injury time points: day 1, day 7, and weekly thereafter up to day 56 (specifically on days 14, 28, and 56). BMS ranged from 0 (complete paraplegia) to 9 (normal function).

### 5.7 MEP

MEP were assessed via electromyography on post‐injury day 56. A stimulating electrode was positioned over the scalp region corresponding to the motor cortex, while a recording electrode was inserted into the gastrocnemius muscle. A reference electrode was placed in the distal tendon of the same hindlimb, and a ground electrode was subcutaneously implanted in the adjacent skin. Single‐pulse stimuli (5 mA intensity, 0.2 ms duration, 1 Hz frequency) were delivered, and the resulting MEP latency and amplitude were recorded for analysis.

### 5.8 NOR Test

The novel object recognition test was conducted on post‐spinal cord injury day 53 to assess cognitive function. 24 h prior to the formal test, mice were individually placed in the center of the empty open field arena for a 5‐minute habituation session. On the test day, two identical objects were placed along a diagonal of the arena, with each object placed 12 cm from the nearest wall. During training, mice were allowed to freely explore for 5 min and then return to the home cages. 1 h later, memory of the object recognition was tested. Mice were allowed to explore for 5 min in the same arena with one of the familiar objects was randomly replaced with a novel object in the original position. All behaviors were video‐recorded and analyzed using Visutrack software. The recognition index (RI) was calculated as follows: RI  =  T novel / (T familiar  +  T novel).

### 5.9 Y‐Maze Test

The Y‐maze test was performed in a symmetrical Y shaped apparatus with three identical arms (35 cm long, 6 cm wide, 15 cm high). The arms were randomly designated as the start arm(S), novel arm(N), and familiar arm(F), with the central junction defined as the center zone. During the training, the novel arm was blocked by a removable partition. Mice were placed at the distal end of the start arm, facing away from the center, and allowed to freely explore the two accessible arms for 5 min. 1 h later, mice were permitted to explore all three arms freely for 5 min with the partition removed. All sessions were video recorded and analyzed using Visutrack software (version 3.0, X Ruan Information Technology Co., Ltd). The time spent in and the number of entries into each arm were recorded and the percentage of time spent in the novel arm and the percentage of entries into the novel arm were calculated (%).

### 5.10 Tissue Collection

Two hours after intracisternal magna injection of the OVA‐647 tracer, mice were deeply anesthetized and transcardially perfused. For histological analysis, mice were perfused with ice‐cold phosphate‐buffered saline (PBS) followed by 4% paraformaldehyde (PFA). dCLNs were dissected and placed in 4% PFA. After removing the scalp and muscles, the skull was opened, and the whole brain was extracted and fixed in 4% PFA for 24 h at 4°C. Following this, the whole‐mount meninges were carefully stripped from the skullcap. The collected meningeal tissues were subsequently stored in PBS at 4°C for later analysis. The fixed brain and dCLNs tissues were cryoprotected by immersion in graded sucrose solutions (15%, 25%, and 30% in sequence) until they sank, followed by embedding in OCT compound and subsequent storage at −80°C. Brain sections (100 or 30 µm) were cut using a Leica cryostat and stored in PBS containing 0.2% sodium azide at 4°C. dCLNs sections (30 µm) were mounted on adhesive slides and kept at −20°C. For molecular analysis and RNA‐seq, mice were perfused with ice‐cold PBS only. dCLNs and the skullcap were dissected and fixed in 4% PFA for 24 h at 4°C. Then, the whole‐mount meninges were carefully stripped from the skullcap and stored in PBS. The motor cortex and hippocampus were rapidly dissected, flash‐frozen in liquid nitrogen, and stored at −80°C for subsequent use.

### 5.11 dCLNs Imaging

The bilateral dCLNs were harvested, and fluorescence imaging was performed using a small animal in vivo imaging system to evaluate the total tracer accumulation intensity in the bilateral dCLNs of each mouse. The dCLNs were stored at 4°C for subsequent dehydration and sectioning.

### 5.12 RNA Extraction and qPCR

Total RNA was extracted from meninges using TRIzol reagent (Invitrogen, USA) according to the manufacturer's instructions. RNA concentration and purity were determined spectrophotometrically (NanoDrop, Thermo Fisher Scientific, USA) based on the absorbance ratios at 260/280 nm. qPCR was performed on a real‐time PCR system using the HiScript II One Step qRT‐PCR SYBR Green Kit (Q221, Vazyme, China) following the manufacturer's protocol. Each 10 µL reaction mixture contained 5 µL of 2× One Step SYBR Green Mix, 0.5 µL of One Step SYBR Green Enzyme Mix, 0.2 µL each of forward and reverse primers (10 µm), 1 µL of RNA template, and 3.1 µL of RNase‐free water. All reactions were performed in triplicate. Gene expression levels were normalized to the endogenous reference gene Gapdh and analyzed using the comparative 2‐ΔΔCt method. The primer sequences used in this study are listed in Table S1.

### 5.13 ELISA

Tissue samples of the cerebral cortex and hippocampus were homogenized to prepare protein suspensions. The concentrations of TNF‐α (BMS607, Invitrogen) and IL‐1β (BMS6002, Invitrogen) were measured using commercial ELISA kits according to the manufacturer's instructions. Briefly, 100 µL of each sample or serially diluted standard was added to the corresponding wells of the pre‐coated plate. After incubation and washing steps, specific antibodies, enzyme conjugates, and chromogenic substrates were sequentially added following the kit protocol. The absorbance of each well was measured at 450 nm using a microplate reader. A standard curve was plotted based on the known concentrations of the standards and their corresponding absorbance values. The concentrations of target cytokines in the samples were then calculated by interpolating their absorbance values into the standard curve equation.

### 5.14 Immunofluorescence Staining

Samples were rinsed three times with PBS (5 min each), permeabilized with 0.3% Triton X‐100 for 30 min, and blocked with 5% BSA for 1 h at room temperature. The sections were then incubated overnight at 4°C with primary antibodies against LYVE‐1(1:500, 67538, Cell Signaling Technology, USA; 14‐0443‐82, Invitrogen, USA), Iba1(1:1000, 011–27991, Wako, Japan), DCX(1:400, 56130, Cell Signaling Technology, USA), Ltbr(1:200, 20331‐1, Proteintech, China), Following three washes with PBS, the sections were incubated with the corresponding secondary antibody: Alexa Fluor 488‐conjugated donkey anti‐rabbit(1:500, ab150073, Abcam, UK), Alexa Fluor 594‐conjugated donkey anti‐goat(1:500, ab150132, Abcam, UK), Alexa Fluor 488‐conjugated donkey anti‐rat(1:500, ab150153, Abcam, UK) for 1 h at room temperature. After a final rinse, the slides were counterstained with Vectashield mounting medium containing DAPI to label cell nuclei. For dura whole mounts, similar blocking and staining procedures were conducted in 24‐well plates with gentle agitation. The isolated dura mater was carefully flattened on a glass slide, with excess moisture removed. Fluorescent images were acquired using a confocal microscope (Zeiss LSM 880, Germany). Quantification of fluorescent area, cell counts, or intensity was performed using ImageJ software. For each animal, three to five representative sections or fields of view were analyzed.

### 5.15 Nissl Staining

Following perfusion and fixation, the brain tissue was carefully dissected and post‐fixed overnight in a 4% paraformaldehyde solution. The specimens were then dehydrated through a graded series of ethanol, cleared in xylene, and embedded in paraffin blocks according to standard histological procedures. Coronal sections were serially cut at a thickness of 5 µm using a rotary microtome. After deparaffinization and rehydration, the sections were stained with a Nissl staining solution strictly following the manufacturer's instructions (G1086, Servicebio). Following the staining procedure, the sections were dehydrated through graded ethanol, cleared in xylene, and coverslipped with neutral balsam. After complete drying, images of the target brain regions were captured under a light microscope for subsequent analysis. The number of positive neurons was quantitatively analyzed using image analysis software.

### 5.16 Whole Dura scRNA‐seq

Fresh dura mater was harvested from three mice and pooled as one sample, then stored in sCelLiveTM Tissue Preservation Solution (Singleron, China) on ice within 30 min after surgery. Single‐cell suspensions were prepared from the dura mater following the manufacturer's instructions of the tissue dissociation kit (1020067, Singleron, China). Cell viability was assessed using Trypan blue staining. scRNA‐seq libraries were constructed using the GEXSCOPE Single Cell RNA Library Kits (Singleron) according to the manufacturer's protocol [44]. Individual libraries were diluted to 4 nm, pooled, and sequenced on the Illumina NovaSeq 6000 platform with 150 bp paired‐end reads.

Raw sequencing reads were processed to generate gene expression profiles using STARSolo v2.0.7 (Singleron Biotechnologies) with default parameters. Quality control, dimensionality reduction, and clustering were performed using Scanpy v1.9.3 within Python 3.10. For each sample, the expression matrix was filtered according to the following criteria: (1) cells with fewer than 200 genes or with gene counts in the top 2% were excluded; (2) cells with UMI counts in the top 2% were excluded; (3) cells with mitochondrial content exceeding 15% were excluded; (4) genes detected in fewer than five cells were excluded. After filtering, 69 877 cells were retained for downstream analyses, with an average of 1409 genes and 3650 UMIs per cell. The raw count matrix was normalized by total counts per cell and log‐transformed. The top 2000 variable genes were selected using the flavor parameter set to Seurat v3. Principal component analysis (PCA) was performed on the scaled variable gene matrix, and the top 18 principal components were used for clustering and dimensionality reduction. Cells were partitioned into 33 clusters using the Louvain algorithm with a resolution parameter of 1.2. Clustering results were visualized using UMAP. Batch effects across samples were corrected using Harmony (v1.0). Prior to Harmony integration, PCA was performed on the top 2000 highly variable genes. The top 18 principal components (PCs) were used as input to Harmony, with the batch assignment variable set to sample_id. Harmony was run with the following default parameters: ridge regression penalty lambda = 1, diversity clustering penalty theta = 2, and maximum Harmony iterations max.iter.harmony = 10. The Harmony‐corrected embeddings were subsequently used for downstream dimensionality reduction using UMAP and unsupervised clustering.

Differentially expressed genes (DEGs) were identified using the scanpy.tl.rank_genes_groups function. The Wilcoxon rank‐sum test (method = ‘wilcoxon’) was used to compare gene expression distributions between groups, as it is a non‐parametric test robust to the sparsity and non‐normality of scRNA‐seq data. Statistical testing was performed on the normalized, log1p‐transformed expression matrix stored in adata.X (use_raw = False), and all genes were evaluated without pre‐selection (n_genes = None). P‐values were adjusted for multiple hypothesis testing using the Benjamini‐Hochberg procedure (corr_method = ‘benjamini‐hochberg’) to control the false discovery rate (FDR). To ensure biological relevance and minimize false positives from low‐abundance genes or dropout events, we applied stringent post‐hoc filtering criteria: (1) a gene was required to be expressed in more than 10% of cells in at least one of the two compared groups (i.e., percentage of cells expressing the gene > 10% in either the test or reference group), which excludes genes detected only in rare cell subsets or driven by technical noise; (2) the average log_2_ fold change (logFC) was required to exceed 0.25, calculated as the difference in mean log‐expression between the two groups, ensuring that only genes with substantial expression differences were retained; and (3) the Benjamini‐Hochberg adjusted *p*‐value (FDR) was required to be less than 0.05.

Cell type annotation was performed based on the expression of canonical markers from the reference database SynEcoSysTM (Singleron Biotechnology), which integrates cell type markers from CellMakerDB, PanglaoDB, and recently published literature.

To investigate the potential functions of lymphatic endothelial cells, GO and KEGG pathway enrichment analyses were performed using the clusterProfiler R package (v3.16.1). Pathways with an adjusted *p*‐value < 0.05 were considered significantly enriched, and selected pathways were visualized as bar plots.

### 5.17 Sorting of Meningeal LECs

Mice were deeply anesthetized and transcardially perfused with ice‐cold PBS. Meningeal LECs were collected according to a previously reported protocol with modifications [18, 45]. The dura mater was carefully dissected and minced in DMEM containing 2% fetal bovine serum (FBS), 1% penicillin‐streptomycin, 1% sodium pyruvate,1% l‐glutamine, 1% sodium pyruvate, 1% non‐essential amino acids (Gibco, USA). The tissue was then digested in DMEM supplemented with 1 mg/mL collagenase VIII and 0.5 mg/mL DNase I at 37°C for 30 min. Following digestion, the meningeal tissue was filtered through a 70 µm cell strainer to obtain a single‐cell suspension. Each sample consisted of pooled cell suspensions derived from 10 meninges. The cell suspension was centrifuged at 280 g for 10 min at 4°C and resuspended in ice‐cold FACS buffer. Subsequently, cells were incubated with the following antibody dilutions for 30 min at 4°C: anti‐CD45‐BB515 (1:200, 564590, BD Biosciences, USA), anti‐CD31‐Alexa Fluor 647 (1:200, 563606, BD Biosciences, USA), and anti‐podoplanin‐PE (1:200, 12‐5381‐82, eBioscience, USA). Finally, DAPI was added for 5 min, after which cells were washed and resuspended in ice‐cold FACS buffer. Single cells were gated based on pulse width using side scatter and forward scatter parameters. Viable cells were identified by negative DAPI staining. Subsequently, DAPI^−^CD45^−^CD31^+^PDPN^+^ cells were gated and sorted using a flow cytometer (BD FACSAria III, BD Biosciences, USA). Sorted target cells were dispensed in droplets at a volume of 1 µL and deposited into PCR tubes containing 5 µL of lysis buffer. Samples were temporarily stored in liquid nitrogen pending further analysis.

### 5.18 Library Preparation and RNA‐seq

All RNA sample processing, cDNA library construction, and RNA‐seq were performed by Lianchuan Biotechnology Co., Ltd (Hangzhou, China). cDNA libraries were constructed from total RNA isolated from three types of samples: sorted meningeal LECs from mice in the spinal cord injury (SCI) and sham‐operated groups, cerebral cortex and hippocampal tissues from the same mouse groups, and hLECs. All libraries were sequenced on an Illumina Novaseq 6000 platform using a 2 × 150 bp paired‐end strategy. Raw reads were filtered and quality‐controlled using Cutadapt (v1.9) to obtain high‐quality clean reads by removing reads containing adapter sequences, polyA/polyG stretches, reads with >5% unknown nucleotides (N), and reads with >20% low‐quality bases (Q ≤ 20). Sequencing quality was then evaluated using FastQC (v0.11.9) by assessing Q20, Q30, and GC content of the clean reads. A total of G bp of valid paired‐end data were obtained after quality control. Clean reads were mapped to the reference genome using HISAT2 (v2.2.1), which allowed up to 2 mismatches and multiple alignments per read, and optimized alignment using a database of potential splice junctions. Transcript assembly was performed with StringTie (v2.1.6) under default parameters. All sample transcriptomes were then merged using gffcompare (v0.9.8) to reconstruct a comprehensive transcriptome. Expression levels of mRNAs were quantified as fragments per kilobase of transcript per million mapped reads (FPKM) using StringTie and ballgown. DEGs between groups were identified using DESeq2, with thresholds of FDR < 0.05 and |log_2_FC| ≥ 1. GO functional enrichment and KEGG pathway enrichment analyses were subsequently performed on DEGs. GSEA (v4.1.0) with the MSigDB database was further applied to detect significantly enriched gene sets. Genes were ranked using the Signal2Noise normalization method, and enrichment was calculated with default parameters. Terms or pathways with |NES| > 1, NOM *p*‐value <0.05, and FDR Q‐value <0.25 were considered significantly different between groups.

### 5.19 hLECs Culture

hLECs was purchased from Yifeng Biosciences (2500, Shanghai, China) and cultured in endothelial cell conditioned medium (1001, ScienCell, USA) at 37°C with 5% CO_2_. The culture medium was renewed every 2 to 3 days until subsequent experimental interventions.

### 5.20 Lentivirus Transfection

hLECs were seeded into sixwell plates at a density of 1 × 10^5^ cells per well 24 h prior to transduction to achieve 40%‐50% confluence at the time of infection. On the day of transduction, the culture medium was replaced with fresh complete medium containing 5 µg/mL polybrene to enhance transduction efficiency. Lentiviral particles for *Ltbr* overexpression and knockdown (Genechem Co., Ltd, China) were added to the cells at a multiplicity of infection (MOI) of 10, which was optimized in preliminary experiments. Cells were incubated with the virus for 12 h, after which the medium was replaced with fresh complete medium to remove residual viral particles. Cells were then cultured for an additional 48 h before subsequent experiments.

### 5.21 Cell Proliferation Assay

Two hours prior to staining, cells were incubated with 10 µmol/L EdU (C0071S, Beyotime, China) at 37°C for 2 h. After EdU labeling, cells were fixed with 4% paraformaldehyde at room temperature for 15 min and permeabilized with 0.3% Triton X‐100 for 10 min. Subsequently, the Click‐iT reaction cocktail containing Alexa Fluor 488 azide was added and incubated for 30 min at room temperature protected from light. Nuclei were counterstained with DAPI (1:1000) for 5 min. Stained cells were visualized under a fluorescence microscope. At least three random fields per sample were selected, and the proliferation rate was calculated as the percentage of EdU positive cells relative to the total number of DAPI‐stained nuclei.

### 5.22 Wound Healing Assay

Transfected cells were seeded into 12‐well plates at a density of 1.4 × 10^5^ cells per well and cultured in endothelial cell conditioned medium until reaching 100% confluence. A linear scratch was then made across the cell monolayer using a sterile 200 µL plastic pipette tip. Detached cells were gently removed by washing with PBS, and the culture medium was replaced with low‐serum medium containing 1% FBS without growth factors. Images were captured under an inverted microscope at 0 and 12 h post‐scratching, and the wound healing area was quantified using ImageJ software.

### 5.23 Tube Formation Experiment

Matrigel (354248, Corning, USA) was thawed on ice at 4°C overnight. A total of 50 µL of pre‐cooled Matrigel was evenly coated onto the bottom of a pre‐cooled 24‐well plate, taking care to avoid bubble formation. The coated plate was then incubated at 37°C for 30 min to allow the Matrigel to solidify. Subsequently, transfected cells were harvested and seeded at a density of 7 × 10^4^ cells per well into the pre‐coated 24‐well plate, and cultured at 37°C in a 5% CO_2_ incubator for 12 h. After incubation, the culture medium was aspirated, and the cells were gently washed with PBS. The cells were then stained with Calcein‐AM (C542, Beyotime, China) for 10 min at 37°C in the dark. Following staining, cells were washed with PBS and immediately visualized and photographed under an inverted fluorescence microscope. Tubule length and the number of branch points were quantitatively analyzed using the angiogenesis analyzer plugin for ImageJ software. All image evaluations were performed by researchers blinded to the experimental groups.

### 5.24 GDC‐0941 Intervention in hLECs

After successful lentiviral transduction, cells were seeded in sixwell plates at 1 × 10^5^ cells per well and cultured under standard conditions until they reached approximately 80% confluence. The PI3K inhibitor GDC‐0941 (pictilisib; HY‐50094, MedChemExpress, USA) was prepared as a stock solution in DMSO and diluted with complete culture medium immediately before treatment. The working concentration of GDC‐0941 (0.5 µm) was selected based on the concentration–response relationship determined by the Cell Counting Kit‐8 assay and the corresponding IC50 analysis. Cells were assigned to four treatment groups comprising Ltbr overexpression (‐/+) and GDC‐0941 treatment (‐/+): LV‐nc + vehicle, LV‐nc + GDC‐0941, LV‐Ltbr + vehicle, and LV‐Ltbr + GDC‐0941. Cells in the inhibitor‐treated groups were exposed to 0.5 µm GDC‐0941 for 24 h, whereas the corresponding control groups received an equivalent volume of vehicle. The final vehicle concentration was maintained identical among all experimental groups.

### 5.25 Western Blotting

After 24 h of intervention, the culture medium was removed, and cells were washed three times with PBS. Cells were subsequently harvested for total protein extraction using RIPA supplemented with protease inhibitors and phosphatase inhibitors. Cell lysates were incubated on ice for 30 min and subsequently centrifuged at 12 000 rpm for 15 min at 4°C. Following centrifugation, the protein content of the hLECs supernatants was quantified using a BCA Protein Assay Kit (Beyotime, China). Total proteins (30 µg per sample) were subjected to SDS‐PAGE using 10% polyacrylamide gels and subsequently electrotransferred onto PVDF membranes (Millipore, USA). The membranes were blocked with 5% skim milk in TBST for 2 h at room temperature and then incubated at 4°C overnight with primary antibodies against PI3K and phospho‐PI3K (1:1000, AF6241, AF3242, Affinity Biosciences, China), AKT and phospho‐AKT (1:2000, 4619S, 4060S, Cell Signaling Technology, USA), and GAPDH (1:5000, A19056, ABclonal, China). After washing with TBST, the membranes were incubated with HRP‐conjugated secondary antibodies at room temperature for 1 h. Immunoreactive bands were visualized using an enhanced chemiluminescence detection system (Bio‐Rad, USA). Band intensities were quantified using ImageJ software. PI3K and AKT phosphorylation levels were evaluated as the p‐PI3K/PI3K and p‐AKT/AKT ratios, respectively.

### 5.26 Statistical Analysis

All statistical analyses were performed using GraphPad Prism 9.0. Data are presented as mean ± SEM. The number of independent biological replicates (n) for each experiment is specified in the Figure legends. Before parametric testing, the normal distribution and homogeneity of variances of data were assessed. For comparisons between two groups, statistical significance was determined using an unpaired two‐tailed Student's t‐test. For comparisons multiple groups, statistical significance was determined using one‐way analysis of variance (ANOVA) followed by Dunnett's or Tukey's multiple comparisons test. Repeated measures ANOVA was performed to analyze the BMS scores over time. Statistical significance was defined as *p*<0.05.

## Author Contributions

**Yi Chen**, **Yu Tang** and **Chengpeng Hu**: conceptualization, methodology, data curation, formal analysis, writing – original draft, writing – review & editing. **Xiaoyan Duan**, **Lingxia Min** and **Boya Cong**: investigation, methodology, validation. **Chao Ma**, **Mingliang Tan**, **Ya Luo**: methodology, software, visualization. **Pengfei Wei**, **Minpeng Xu**: supervision, software. **Zhou Feng** and **Jingming Hou**: conceptualization, methodology, writing – review & editing, project administration.

## Funding

This work was supported by grants from the National Natural Science Foundation of China (Nos. 82301569 and 82472607) and Chongqing High‐end Young‐and Middle‐aged Medical Talent Project (No. YXGD202460); Chongqing Outstanding Medical Youth Talent Project (No. YXQN202417).

## Conflicts of Interest

The authors declare no conflicts of interest.

## Supporting information




**Supporting File**: advs78206‐sup‐0001‐SuppMat.docx.

## Data Availability

The data supporting the findings of this study are available from the corresponding author.

## References

[1] J. Hou , Z. Xiang , R. Yan , et al., “Motor Recovery at 6 Months After Admission is Related to Structural and Functional Reorganization of the Spine and Brain in Patients With Spinal Cord Injury,” Human Brain Mapping 37, no. 6 (2016): 2195–2209, 10.1002/hbm.23163. PMC6867385 26936834

[2] O. V. Alcantar‐Garibay , D. Incontri‐Abraham , and A. Ibarra , “Spinal Cord Injury‐Induced Cognitive Impairment: A Narrative Review,” Neural Regeneration Research 17, no. 12 (2022): 2649–2654, 10.4103/1673-5374.339475. PMC9165403 35662196

[3] H. Liu , Y. Ni , H. Xie , et al., “The Network and Interactive Pattern of Social Adjustment and Psychological Symptoms in Patients With Spinal Cord Injury: A Network Analysis,” BMC Psychology 13, no. 1 (2025): 774, 10.1186/s40359-025-03105-0. PMC12247250 40646648

[4] D. Sandalic , A. Craig , Y. Tran , et al., “Cognitive Impairment in Individuals With Spinal Cord Injury,” Neurology 99, no. 16 (2022): e1779–e1790, 10.1212/WNL.0000000000200957. 35985827

[5] X. Hu , W. Xu , Y. Ren , et al., “Spinal Cord Injury: Molecular Mechanisms and Therapeutic Interventions,” Signal Transduction and Targeted Therapy 8, no. 1 (2023): 245, 10.1038/s41392-023-01477-6. PMC10291001 37357239

[6] J.‐M. Hou , R.‐B. Yan , Z.‐M. Xiang , et al., “Brain Sensorimotor System Atrophy During the Early Stage of Spinal Cord Injury in Humans,” Neuroscience 266 (2014): 208–215, 10.1016/j.neuroscience.2014.02.013. 24561217

[7] G. Ziegler , P. Grabher , A. Thompson , et al., “Progressive Neurodegeneration Following Spinal Cord Injury,” Neurology 90, no. 14 (2018): e1257–e1266, 10.1212/WNL.0000000000005258. PMC5890610 29514946

[8] S. Schading , G. David , T. Max Emmenegger , et al., “Dynamics of Progressive Degeneration of Major Spinal Pathways Following Spinal Cord Injury: A Longitudinal Study,” NeuroImage: Clinical 37 (2023): 103339, 10.1016/j.nicl.2023.103339. PMC9939725 36758456

[9] Z. Feng , L. Min , H. Chen , et al., “Iron Overload in the Motor Cortex Induces Neuronal Ferroptosis Following Spinal Cord Injury,” Redox Biology 43 (2021): 101984, 10.1016/j.redox.2021.101984. PMC8105676 33933882

[10] Y. Li , R. M. Ritzel , N. Khan , et al., “Delayed Microglial Depletion After Spinal Cord Injury Reduces Chronic Inflammation and Neurodegeneration in the Brain and Improves Neurological Recovery in Male Mice,” Theranostics 10, no. 25 (2020): 11376–11403, 10.7150/thno.49199. PMC7545988 33052221

[11] X. Hu , Y. Zhang , L. Wang , et al., “Microglial Activation in the Motor Cortex Mediated NLRP3‐Related Neuroinflammation and Neuronal Damage Following Spinal Cord Injury,” Frontiers in Cellular Neuroscience 16 (2022): 956079, 10.3389/fncel.2022.956079. PMC9630363 36339822

[12] I. Jure , A. F. De Nicola , J. M. Encinas , and F. Labombarda , “Spinal Cord Injury Leads to Hippocampal Glial Alterations and Neural Stem Cell Inactivation,” Cellular and Molecular Neurobiology 42, no. 1 (2022): 197–215, 10.1007/s10571-020-00900-8. PMC11441270 32537668

[13] A. Louveau , I. Smirnov , T. J. Keyes , et al., “Structural and Functional Features of Central Nervous System Lymphatic Vessels,” Nature 523, no. 7560 (2015): 337–341, 10.1038/nature14432. PMC4506234 26030524

[14] J. J. Iliff , M. Wang , Y. Liao , et al., “A Paravascular Pathway Facilitates CSF Flow Through the Brain Parenchyma and the Clearance of Interstitial Solutes, Including Amyloid β,” Science Translational Medicine 4, no. 147 (2012): 147ra111, 10.1126/scitranslmed.3003748. PMC3551275 22896675

[15] Q. Zhang , Y. Niu , Y. Li , et al., “Meningeal Lymphatic Drainage: Novel Insights Into Central Nervous System Disease,” Signal Transduction and Targeted Therapy 10, no. 1 (2025): 142, 10.1038/s41392-025-02177-z. PMC12050339 40320416

[16] H. Dong , X. Dai , Y. Zhou , et al., “Enhanced Meningeal Lymphatic Drainage Ameliorates Lipopolysaccharide‐Induced Brain Injury in Aged Mice,” Journal of Neuroinflammation 21, no. 1 (2024): 36, 10.1186/s12974-024-03028-4. PMC10826026 38287311

[17] A. C. Bolte , A. B. Dutta , M. E. Hurt , et al., “Meningeal Lymphatic Dysfunction Exacerbates Traumatic Brain Injury Pathogenesis,” Nature Communications 11, no. 1 (2020): 4524, 10.1038/s41467-020-18113-4. PMC7483525 32913280

[18] S. Da Mesquita , Z. Papadopoulos , T. Dykstra , et al., “Meningeal Lymphatics Affect Microglia Responses and Anti‐Aβ Immunotherapy,” Nature 593, no. 7858 (2021): 255–260, 10.1038/s41586-021-03489-0. PMC8817786 33911285

[19] A. Louveau , J. Herz , M. N. Alme , et al., “CNS Lymphatic Drainage and Neuroinflammation Are Regulated by Meningeal Lymphatic Vasculature,” Nature Neuroscience 21, no. 10 (2018): 1380–1391, 10.1038/s41593-018-0227-9. PMC6214619 30224810

[20] L. S. B. Boisserand , L. H. Geraldo , J. Bouchart , et al., “VEGF‐C Prophylaxis Favors Lymphatic Drainage and Modulates Neuroinflammation in a Stroke Model,” Journal of Experimental Medicine 221, no. 4 (2024): 20221983, 10.1084/jem.20221983. PMC10913814 38442272

[21] X. Mei , J. Xiong , J. Liu , et al., “DHCR7 Promotes Lymph Node Metastasis in Cervical Cancer Through Cholesterol Reprogramming‐Mediated Activation of the KANK4/PI3K/AKT Axis and VEGF‐C Secretion,” Cancer Letters 584 (2024): 216609, 10.1016/j.canlet.2024.216609. 38211648

[22] W. Wang , S. Tang , C. Li , et al., “Specific Brain Morphometric Changes in Spinal Cord Injury: A Voxel‐Based Meta‐Analysis of White and Gray Matter Volume,” Journal of Neurotrauma 36, no. 15 (2019): 2348–2357, 10.1089/neu.2018.6205. 30794041

[23] Z. Wu , K. Feng , J. Huang , et al., “Brain Region Changes Following a Spinal Cord Injury,” Neurochemistry International 174 (2024): 105696, 10.1016/j.neuint.2024.105696. 38354751

[24] P. Freund , N. Weiskopf , N. S. Ward , et al., “Disability, Atrophy and Cortical Reorganization Following Spinal Cord Injury,” Brain 134, no. Pt 6 (2011): 1610–1622, 10.1093/brain/awr093. PMC3102242 21586596

[25] Q. Chen , W. Zheng , X. Chen , et al., “Whether Visual‐Related Structural and Functional Changes Occur in Brain of Patients With Acute Incomplete Cervical Cord Injury: A Multimodal Based MRI Study,” Neuroscience 393 (2018): 284–294, 10.1016/j.neuroscience.2018.10.014. 30326291

[26] W.‐Q. Zhang , J.‐P. Wu , L. Yu , et al., “Association Between Spinal Cord Injury and Cognitive Impairment, and the Mediating Role of Inflammation,” Journal of Craniofacial Surgery 36, no. 6 (2025): e799–e803, 10.1097/SCS.0000000000011593. 40569607

[27] Z. Lei , B. Krishnamachary , N. Z. Khan , et al., “Spinal Cord Injury Disrupts Plasma Extracellular Vesicles Cargoes Leading to Neuroinflammation in the Brain and Neurological Dysfunction in Aged Male Mice,” Brain, Behavior, and Immunity 120 (2024): 584–603, 10.1016/j.bbi.2024.07.005. PMC11269008 38986724

[28] X. Guo , Y. Zhu , S. Gao , Y. Wu , and Y. Hong , “Meningeal Macrophages Regulate Fibroblasts to Influence Meningeal Lymphatic Function Following Traumatic Brain Injury,” Theranostics 16, no. 10 (2026): 5185–5203, 10.7150/thno.130263. PMC13080450 41993613

[29] X.‐B. Ding , X.‐X. Wang , D.‐H. Xia , et al., “Impaired Meningeal Lymphatic Drainage in Patients With Idiopathic Parkinson's Disease,” Nature Medicine 27, no. 3 (2021): 411–418, 10.1038/s41591-020-01198-1. 33462448

[30] B. Zhu , C. Liu , M. Luo , et al., “Spatiotemporal Dynamic Changes of Meningeal Microenvironment Influence Meningeal Lymphatic Function Following Subarachnoid Hemorrhage: From Inflammatory Response to Tissue Remodeling,” Journal of Neuroinflammation 22, no. 1 (2025): 131, 10.1186/s12974-025-03460-0. PMC12083004 40380229

[31] T. Taoka , Y. Masutani , H. Kawai , et al., “Evaluation of Glymphatic System Activity With the Diffusion MR Technique: Diffusion Tensor Image Analysis Along the Perivascular Space (DTI‐ALPS) in Alzheimer's Disease Cases,” Japanese Journal of Radiology 35, no. 4 (2017): 172–178, 10.1007/s11604-017-0617-z. 28197821

[32] B. Ransmayr , S. K. Bal , M. Thian , et al., “LTβR Deficiency Causes Lymph Node Aplasia and Impaired B Cell Differentiation,” Science Immunology 9, no. 101 (2024): adq8796, 10.1126/sciimmunol.adq8796. PMC7618087 39576873

[33] P. D. Rennert , D. James , F. Mackay , J. L. Browning , and P. S. Hochman , “Lymph Node Genesis is Induced by Signaling Through the Lymphotoxin β Receptor,” Immunity 9, no. 1 (1998): 71–79, 10.1016/s1074-7613(00)80589-0. 9697837

[34] C. C. Brinkman , D. Iwami , M. K. Hritzo , et al., “Treg Engage Lymphotoxin Beta Receptor for Afferent Lymphatic Transendothelial Migration,” Nature Communications 7 (2016): 12021, 10.1038/ncomms12021. PMC4919545 27323847

[35] J. Du , P. Liu , Y. Zhou , et al., “The Mechanosensory Channel PIEZO1 Functions Upstream of Angiopoietin/TIE/FOXO1 Signaling in Lymphatic Development,” Journal of Clinical Investigation 134, no. 10 (2024): 176577, 10.1172/JCI176577. PMC11093609 38747287

[36] R. Sato‐Nishiuchi , M. Doiguchi , N. Morooka , and K. Sekiguchi , “Polydom/SVEP1 Binds to Tie1 and Promotes Migration of Lymphatic Endothelial Cells,” Journal of Cell Biology 222, no. 9 (2023): 202208047, 10.1083/jcb.202208047. PMC10281526 37338522

[37] D. Choi , E. Park , J. Choi , et al., “Piezo1 Regulates Meningeal Lymphatic Vessel Drainage and Alleviates Excessive CSF Accumulation,” Nature Neuroscience 27, no. 5 (2024): 913–926, 10.1038/s41593-024-01604-8. PMC11088999 38528202

[38] T. M. Conlon , G. John‐Schuster , D. Heide , et al., “Inhibition of LTβR Signalling Activates WNT‐Induced Regeneration in Lung,” Nature 588, no. 7836 (2020): 151–156, 10.1038/s41586-020-2882-8. PMC7718297 33149305

[39] M. C. Lund , B. H. Clausen , R. Brambilla , and K. L. Lambertsen , “The Role of Tumor Necrosis Factor Following Spinal Cord Injury: A Systematic Review,” Cellular and Molecular Neurobiology 43, no. 3 (2023): 925–950, 10.1007/s10571-022-01229-0. PMC11414445 35604578

[40] T. Qin , Y. Qin , Y. Jin , et al., “Extracellular Vesicle‐Mediated Spinal Cord‐Brain Crosstalk Induces Hippocampal Neurogenesis Impairment and Cognitive Deficits Post‐Spinal Cord Injury,” Theranostics 15, no. 15 (2025): 7584–7606, 10.7150/thno.110560. PMC12316029 40756360

[41] X. Liu , G. Barisano , X. Shao , et al., “Cross‐Vendor Test‐Retest Validation of Diffusion Tensor Image Analysis Along the Perivascular Space (DTI‐ALPS) for Evaluating Glymphatic System Function,” Aging and Disease 15, no. 4 (2024): 1885–1898, 10.14336/AD.2023.0321-2. PMC11272201 37307817

[42] O. G. Bhalala , L. Pan , H. North , T. McGuire , and J. A. Kessler , “Generation of Mouse Spinal Cord Injury,” Bio‐Protocol 3, no. 17 (2013): 886, 10.21769/bioprotoc.886. PMC4978511 27517060

[43] M. Wang , C. Yan , X. Li , et al., “Non‐Invasive Modulation of Meningeal Lymphatics Ameliorates Ageing and Alzheimer's Disease‐Associated Pathology and Cognition in Mice,” Nature Communications 15, no. 1 (2024): 1453, 10.1038/s41467-024-45656-7. PMC10873306 38365740

[44] B. Dura , J.‐Y. Choi , K. Zhang , et al., “scFTD‐seq: Freeze‐Thaw Lysis Based, Portable Approach Toward Highly Distributed Single‐Cell 3′ mRNA Profiling,” Nucleic Acids Research 47, no. 3 (2019): 16, 10.1093/nar/gky1173. PMC6379653 30462277

[45] J. Rustenhoven , A. Drieu , T. Mamuladze , et al., “Functional Characterization of the Dural Sinuses as a Neuroimmune Interface,” Cell 184, no. 4 (2021): 1000–1016.e27, 10.1016/j.cell.2020.12.040. PMC8487654 33508229

