# Supplementary material for: Meningeal Lymphatic LTBR Deficiency Links Spinal Cord Injury to Secondary Brain Pathology
Source: Adv Sci (Weinh). 2026 Oct 11:e78206. Online ahead of print. doi: 10.1002/advs.78206 (PMC13655250; doi:10.1002/advs.78206)
Supplement: Supplementary file 1 [file ADVS-9999-e78206-s001.docx]

Supporting Information

Title: Meningeal Lymphatic LTBR Deficiency Links Spinal Cord Injury to Secondary Brain Pathology

Yi Chen^1^, Yu Tang^1^, Chengpeng Hu^1^, Xiaoyan Duan^1^, LingXia Min^1^, Boya Cong^1^, Chao Ma^1^, Mingliang Tan^1^, Ya Luo^1^, Pengfei Wei^2^, Minpeng Xu^3^, Zhou Feng^1^*, Jingming Hou^1^*

**Figure S1. The effects of SCI on** **neurological deficits and remote secondary brain pathology**

**(A)** Locomotor recovery was quantified using the BMS in both sham and SCI mice across multiple post-injury time points (n=10-12 mice per group). Statistical significance was determined by repeated measures ANOVA. **(B)** Representative MEP traces from sham and SCI mice at 56 dpi. (**C-D)** Quantification of MEP latency and amplitude in sham and SCI mice at 56 dpi (n=8 mice per group). **(E)** Quantification of the object recognition index from the NOR test in sham and SCI mice at 56 dpi (n=10-12 mice per group).  **(F)** Representative Y-maze heatmaps showing exploration patterns of sham and SCI mice at 56 dpi (S: start arm, N: novel arm, F: familiar arm). **(G-H)** Quantification of the number of entries into the novel arm and the duration spent in the novel arm in sham and SCI mice at 56 dpi (n=10-12 mice per group). **(I-J)** TNF-α and IL-1β levels in the cortex and hippocampus of sham and SCI mice at 56 dpi (n = 5 mice per group). **(K-L)** Representative immunofluorescence images and quantification of Iba1⁺ microglia (red) and DAPI (blue) in the cortex and hippocampus of sham and SCI mice at 56 dpi (n = 6 mice per group). Scale bar, 100 μm. **(M-N)** Representative Nissl staining images and quantification of surviving neurons in the cortex and hippocampus of sham and SCI mice at 56 dpi (n = 4 mice per group). Scale bar, 50 μm. **(O-P)** Representative immunofluorescence images and quantification of DCX⁺ neuronal progenitors (green) and DAPI(blue) in the hippocampal dentate gyrus of sham and SCI mice at 56 dpi (n = 6 mice per group), Scale bar, 200 μm. **(Q-R)** KEGG pathway enrichment analysis of DEGs in the motor cortex and hippocampus of SCI mice compared to sham group at 56 dpi (n = 4 mice per group). **(S-T)** Heatmap showing the relative expression levels of DEGs involved in the NF‑κB signaling and IL‑17 signaling pathways in the motor cortex and hippocampus (n = 4 mice per group). Statistical significance was determined using an unpaired two-tailed Student’s t-test. Data are presented as mean ± SEM. **P* < 0.05, ***P* < 0.01, ****P* < 0.001, *****P* < 0.0001.

**Figure S2. Transcriptomic profiling of motor cortex and hippocampus after SCI**

**(A-B)** Volcano plots illustrating DEGs in the motor cortex and hippocampus of SCI mice relative to sham controls (n = 4 mice per group). Red dots represent significantly upregulated genes, blue dots represent significantly downregulated genes, and gray dots represent genes with no significant expression change. **(C-D)** GO enrichment scatter plots for the motor cortex and hippocampus, showing the top enriched biological processes in SCI mice (n = 4 mice per group). The size of each dot corresponds to the number of enriched genes, and the color gradient represents the statistical significance. **(E-F)** GSEA plots demonstrating the enrichment of representative gene sets for the motor cortex and hippocampus in SCI versus sham mice (n = 4 mice per group).

**Figure S3. The effect of mLVs ablation on lymphatic function in SCI mice**

**(A)** Schematic timeline of the experimental design. **(B-C)** In vivo fluorescence imaging and quantitative analysis of tracer (OVA-647) accumulation in the dCLNs at 56 dpi, in Visudyne+laser-treated SCI mice compared with Visudyne SCI controls (n=6 mice per group). **(D-E)** Representative immunofluorescence staining and quantitative analysis of OVA-647-positive area in dCLNs sections (n=6 mice per group). Scale bar, 200 μm. **(F-G)** Representative LYVE-1 immunofluorescence staining of the dura mater and quantitative analysis of LYVE-1^+^ vessel area fraction (n=6 mice per group). Scale bar, 1000 μm. **(H-I)** Representative whole-brain fluorescence images and quantitative analysis of OVA-647^+^ area fraction in the brain parenchyma (n=3 mice per group). Scale bar, 1000 mm. Statistical significance was determined using an unpaired two-tailed Student’s t-test. Data are presented as mean ± SEM. **P* < 0.05, ***P* < 0.01, ****P* < 0.001.

**Figure S4. mLVs ablation exacerbates SCI-induced neurological deficits and secondary brain pathology**

**(A)** BMS locomotor scores over 56 dpi in Visudyne and Visudyne+laser SCI mice (n=10-12 mice per group). Statistical significance was determined by repeated measures ANOVA. **(B)** Representative MEP traces recorded at 56 dpi. **(C-D)** Quantitative analysis of MEP latency and amplitude (n=10-12 mice per group). **(E)** Object recognition index from the NOR test (n=10-12 mice per group). **(F)** Representative heatmaps of Y-maze exploration. **(G-H)** Quantitative analysis of entries into the novel arm and duration in the novel arm during the Y-maze test (n=10-12 mice per group). **(I-J)** ELISA quantification of TNF-α and IL-1β levels in the cerebral cortex and hippocampus (n=5 mice per group). **(K-L)** Representative immunofluorescence staining and quantitative analysis of Iba1^+^ microglia (red) and DAPI (blue) in the cortex and hippocampus (n=5 mice per group). Scale bar, 50 μm. **(M-N)** Representative Nissl staining and quantitative analysis of surviving neurons in the cortex and hippocampus (n=5 mice per group). Scale bar, 50 μm. **(O-P)** Representative immunofluorescence staining and quantitative analysis of DCX-positive area (green) in the hippocampus, with DAPI (blue) (n=5 mice per group). Scale bar, 200 μm. Statistical significance was determined using an unpaired two-tailed Student’s t-test. Data are presented as mean ± SEM. **P* < 0.05, ***P* < 0.01, ****P* < 0.001. *****P* < 0.0001

**Figure S5. Quality control, LECs marker expression, and predicted cell-cell communication in the dura mater scRNA-seq dataset.**

**(A)** Distribution of the Percent_mt across individual samples (n = 3 independent biological replicates per group; each replicate pooled from three mice). **(B)** Distribution of nCount_RNA across individual samples (n = 3 independent biological replicates per group; each replicate pooled from three mice). **(C)** UMAP feature plots showing the expression of the LECs-associated markers *Flt4*, *Lyve1*, and *Prox1*. **(D)** Heatmap showing predicted cell-cell communication strength between LECs and major dural cell populations, including immune cell populations.

**Figure S6. Transcriptomic analysis of** **meningeal LECs reveals no significant enrichment of the canonical VEGF-C signaling pathway in SCI mice**

**(A)** GSEA plot demonstrating the enrichment profile of the VEGF-C signaling pathway gene set in SCI versus sham meningeal LECs (n=3 independent biological replicates per group; each replicate pooled from six mice). **(B-E)** Violin plots showing the expression levels of key VEGF-C signaling pathway-related genes, including *Vegf-c*, *Nrp2*, *Calm2*, and *Vegf-d*, in meningeal LECs isolated from sham and SCI groups.

**Figure S7. LTBR deficiency does not alter basal meningeal lymphatic drainage or lymphatic vessel coverage in mice without SCI.**

**(A)** Representative whole-mount fluorescence images of the meninges from control (AAV9-nc) and LTBR-deficient (AAV9-shLtbr) mice following intracisternal administration of OVA-647. OVA-647 is shown in red, LYVE-1 in green, and DAPI in blue. Scale bar, 200 μm. **(B)** Quantification of the OVA-647^+^ area fraction in the meninges (n=5 mice per group). **(C)** Representative whole-mount fluorescence images showing LYVE-1^+^ meningeal lymphatic vessels in control and LTBR-deficient mice. LYVE-1 is shown in red and DAPI in blue, Scale bar, 1000 μm. **(D)** Quantification of the LYVE-1^+^ vessel area fraction (n=5 mice per group). Statistical significance was determined using an unpaired two-tailed Student’s t-test. Data are presented as mean ± SEM. ns, not significant.

**Figure S8. Generation and validation of *Ltbr* gene modification in LECs**

**(A)** PCR-based genotyping confirmation of *Ltbr* gene modification (n=15 mice per group). **(B)** Representative immunofluorescence staining images of LTBR (red), LYVE-1(green), and DAPI (blue) in the dura mater of Ltbr^CKI/+^ and Ltbr^CKI/+^; Prox1-Cre mice. **(C)** Quantitative comparison of LTBR/LYVE-1 co-localization area (n=3 mice per group). Statistical significance was determined using an unpaired two-tailed Student’s t-test. Data are presented as mean ± SEM, **P* < 0.05.

**Figure S9. Pharmacological inhibition of PI3K attenuates LTBR-mediated LECs responses.**
**(A)** Dose-response analysis of GDC-0941 in hLECs. **(B)** Representative Western blots showing p-PI3K, total PI3K, p-AKT, total AKT, and GAPDH in hLECs under the indicated conditions(n=3 independent biological replicates). **(C-D)** Quantification of the p-PI3K/PI3K and p-AKT/AKT ratios, respectively. **(E-F)** Representative images and quantification of EdU staining (green) and DAPI(blue) in hLECs(n=5 independent biological replicates). **(G-H)** Representative images and quantification of scratch wound healing assays(n=5 independent biological replicates). **(I-J)** Representative images and quantification of tube formation assays based on total tube length (n=5 independent biological replicates). Statistical significance was determined by one-way ANOVA followed by Tukey’s multiple comparisons test. Data are presented as mean ± SEM. **P* < 0.05, ***P* < 0.01, ****P* < 0.001, *****P* < 0.0001.

**Table S1. Primer sequences used for qPCR**

| Name |  | Primers for qPCR (5′-3′) |
| --- | --- | --- |
| m-*Gapdh* | F | TGTGTCCGTCGTGGATCTGA |
|  | R | TTGCTGTTGAAGTCGCAGGAG |
| m-*Vegfc* | F | TCTGTGTCCAGCGTAGATGAG |
|  | R | GTCCCCTGTCCTGGTATTGAG |
| m-*Ltbr* | F | GCGAGTTTGTCTTTGCGGTAT |
|  | R | GGAGAGATGGTTCCAGTGTTCA |
| h-*Gapdh* | F | GCACCGTCAAGGCTGAGAAC |
|  | R | TGGTGAAGACGCCAGTGGA |
| h-*Ltbr* | F | TCAAAGATGAAGTTGGGAAGGGT |
|  | R | GGGCAGTGGCTCTAATGGAT |
